# Plasmids Shaped the Recent Emergence of the Major Nosocomial Pathogen Enterococcus faecium

**DOI:** 10.1128/mBio.03284-19

**Published:** 2020-02-11

**Authors:** S. Arredondo-Alonso, J. Top, A. McNally, S. Puranen, M. Pesonen, J. Pensar, P. Marttinen, J. C. Braat, M. R. C. Rogers, W. van Schaik, S. Kaski, R. J. L. Willems, J. Corander, A. C. Schürch

**Affiliations:** a Department of Medical Microbiology, University Medical Center Utrecht, Utrecht, The Netherlands; b Institute of Microbiology and Infection, University of Birmingham, Birmingham, United Kingdom; c Department of Computer Science, Aalto University, Espoo, Finland; d Department of Mathematics and Statistics, Helsinki Institute of Information Technology (HIIT), University of Helsinki, Helsinki, Finland; e Pathogen Genomics, Wellcome Trust Sanger Institute, Cambridge, United Kingdom; f Department of Biostatistics, University of Oslo, Oslo, Norway; MedImmune

**Keywords:** *Enterococcus faecium*, long-read sequencing, machine learning, nosocomial pathogen, plasmidome, source specificity

## Abstract

Enterococcus faecium is a gut commensal of humans and animals but is also listed on the WHO global priority list of multidrug-resistant pathogens. Many of its antibiotic resistance traits reside on plasmids and have the potential to be disseminated by horizontal gene transfer. Here, we present the first comprehensive population-wide analysis of the pan-plasmidome of a clinically important bacterium, by whole-genome sequence analysis of 1,644 isolates from hospital, commensal, and animal sources of E. faecium. Long-read sequencing on a selection of isolates resulted in the completion of 305 plasmids that exhibited high levels of sequence modularity. We further investigated the entirety of all plasmids of each isolate (plasmidome) using a combination of short-read sequencing and machine-learning classifiers. Clustering of the plasmid sequences unraveled different E. faecium populations with a clear association with hospitalized patient isolates, suggesting different optimal configurations of plasmids in the hospital environment. The characterization of these populations allowed us to identify common mechanisms of plasmid stabilization such as toxin-antitoxin systems and genes exclusively present in particular plasmidome populations exemplified by copper resistance, phosphotransferase systems, or bacteriocin genes potentially involved in niche adaptation. Based on the distribution of k-mer distances between isolates, we concluded that plasmidomes rather than chromosomes are most informative for source specificity of E. faecium.

## Abstract (precis)

Enterococcus faecium is one of the most frequent nosocomial pathogens of hospital-acquired infections. E. faecium has gained resistance against most commonly available antibiotics, most notably, against ampicillin, gentamicin, and vancomycin, which renders infections difficult to treat. Many antibiotic resistance traits, in particular, vancomycin resistance, can be encoded in autonomous and extrachromosomal elements called plasmids. These sequences can be disseminated to other isolates by horizontal gene transfer and confer novel mechanisms to source specificity. In our study, we elucidated the total plasmid content, referred to as the plasmidome, of 1,644 E. faecium isolates by using short- and long-read whole-genome technologies with the combination of a machine-learning classifier. This was fundamental to investigate the full collection of plasmid sequences present in our collection (pan-plasmidome) and to observe the potential transfer of plasmid sequences between E. faecium hosts. We observed that E. faecium isolates from hospitalized patients carried a larger number of plasmid sequences compared to that from other sources, and they elucidated different configurations of plasmidome populations in the hospital environment. We assessed the contribution of different genomic components and observed that plasmid sequences have the highest contribution to source specificity. Our study suggests that E. faecium plasmids are regulated by complex ecological constraints rather than physical interaction between hosts.

## INTRODUCTION

Enterococcus faecium ranks among the most frequent causative agents of hospital-acquired infections, specifically, central-line associated bloodstream infections (1). The burden of disease due to E. faecium is augmented by the fact that E. faecium has acquired resistance against almost all available antibiotics, most notably, against ampicillin, gentamicin, and vancomycin and less frequently against the more recently introduced antibiotics linezolid, daptomycin, and tigecycline (2). Antibiotic resistance, including vancomycin resistance, is not a feature exclusively found among hospitalized patient isolates, as E. faecium isolates from farm animals also contain these resistance traits (3).

Previous whole-genome sequencing (WGS)-based studies split the E. faecium population into two lineages corresponding to a hospital-associated clade (clade A) and a community-associated clade (clade B) (4, 5). Subsequently, clade A was first subdivided into clade A1, mainly represented by clinical isolates, and clade A2, with a majority of animal isolates (6). Recent reports indicated that animal isolates do not form a monophyletic subclade and no longer support the split of clade A isolates into two single subclades (2, 7).

Plasmids can act as vehicles for the transmission of virulence and antimicrobial resistance genes (8). Several mechanisms of plasmid-mediated resistance have been described in E. faecium (9, 10), including glycopeptide resistance caused by the presence of *vanA* and *vanB* gene clusters (Tn*1546* and Tn*1549*, respectively), aminoglycoside resistance caused by the presence of *aac(6′)-Ie-aph(2″)* gene (Tn*5281*), tetracycline resistance mediated by *tet*(M), linezolid resistance due to the presence of *cfr*, *cfr*(B), *optrA*, and *poxtA*, or quinupristin-dalfopristin resistance due to plasmids harboring *vat*(D) and *vat*(E).

Enterococcal plasmids have been conventionally grouped in four main family groups (RepA_N, Inc18, RCR, and Rep_3) based on their sequence homology against known replication initiator proteins (RIP) (11). The presence of conjugation systems and mobilization systems in enterococcal plasmids suggests that horizontal gene transfer (HGT) may act as a major source of DNA mobility between E. faecium hosts (11). Previous attempts to investigate the mobilome and HGT in E. faecium have been restricted to microarray-based studies using custom-designed probes (12).

In this study, we sequenced the genomes of 1,644 clade A isolates from human (hospitalized patients and nonhospitalized persons) and animal (pet, farm, and wild animals) sources using short-read sequencing technology. We elucidated complete plasmid sequences from a representative subset of 62 isolates by long-read sequencing, resulting in 305 complete plasmids. Furthermore, we used a recently developed machine-learning classifier (mlplasmids) to predict the plasmidome of E. faecium isolates with only short-read sequencing data (13). Using this novel genomic tool, we accurately predicted and defined the plasmidome of all isolates that were sequenced as part of this study, which allowed the study of the population pan-plasmidome of E. faecium in terms of plasmid k-mers and gene diversity in the clade A isolates. Our analysis shows that the plasmidome rather than the chromosome of E. faecium is most informative for understanding niche adaptation.

## RESULTS

### Core gene phylogeny confirms distinct clustering of hospitalized patient isolates.

**FIG 1:**
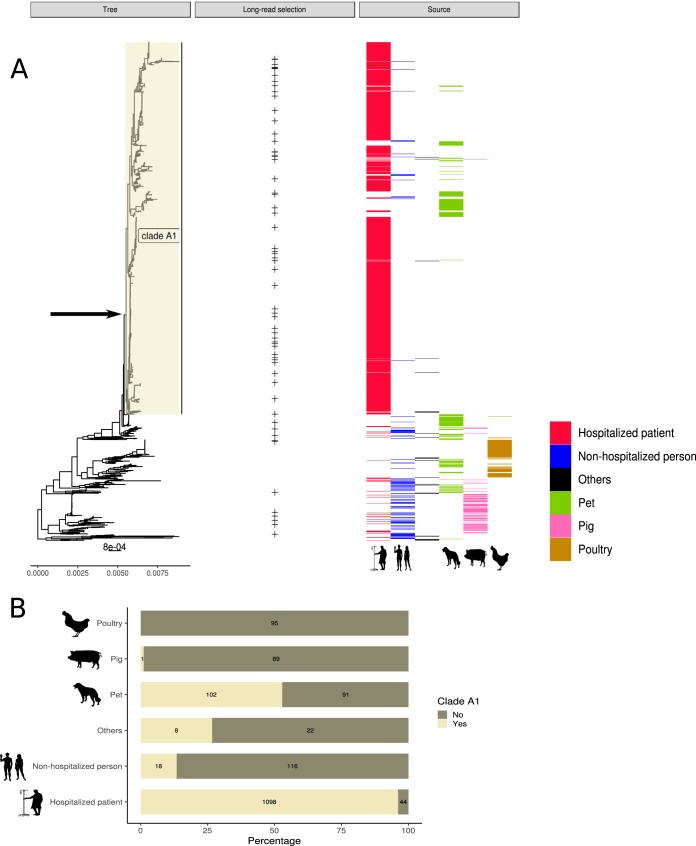
(A) RAxML tree based on 955 E. faecium core genes in 1,644 clade A strains. Isolates selected for long-read sequencing are indicated with + under long-read selection. Isolates were colored based on their isolation source: hospitalized patients (red), nonhospitalized persons (blue), pet (green), pig (pink), poultry (brown), and other sources (black). Arrow in the RAxML tree indicates the internal node 1227 used to split the clade A1 and non-clade A1 isolates. (B) For each isolation source (*x* axis), we specified the count and percentage (*y* axis) of isolates belonging or not to clade A1.

To determine the core genome variability of clade A E. faecium isolates, we constructed a core gene alignment for 1,644 isolates of E. faecium clade A. This alignment was filtered for recombination, and the remaining variable sites were analyzed to classify the 1,644 isolates into (85) sequence clusters (SCs) using hierBAPS (postBNGBAPS.2 group) (see Data Set S1 in the supplemental material). In total, 955 genes (orthologous groups) were used to reconstruct the population phylogeny of our E. faecium collection (Fig. 1A) (https://microreact.org/project/BJKGTJPTQ).

10.1128/mBio.03284-19.9 DATA SET S1 Additional files containing metadata information from the collection of 1,644 E. faecium isolates, hybrid assembly statistics of the 62 long-read sequenced E. faecium isolates, RIP and relaxase searches in the set of 305 complete plasmid sequences, plasmidome population core genes, and eggNOG annotation. Download Data Set S1, XLSX file, 0.4 MB. Copyright © 2020 Arredondo-Alonso et al. 2020 Arredondo-Alonso et al. This content is distributed under the terms of the Creative Commons Attribution 4.0 International license.

In accordance with previous E. faecium population studies, we split the 1,644 E. faecium isolates into clade A1 and non-clade A1 isolates (Fig. 1). Hospitalized patient isolates (1,142) were mostly designated clade A1 (1,098; 96%), representing the most frequent source in this clade (1,098/1,227 [89%]). We also identified clade A1 isolates in nonhospitalized persons (18) and pets (102) (Fig. 1B). Furthermore, pet isolates represented the biggest nonhospital source (78%) present in clade A1 (Fig. 1B). These pet isolates were mainly from dogs from the Netherlands, randomly selected in an unbiased nationwide survey of healthy pet owners with no recent antibiotic usage history. In this survey, cocarriage of vancomycin-resistant E. faecium between owners and dogs was not observed (14).

Human community isolates from nonhospitalized patients were widely dispersed over the phylogenetic tree outside clade A1 (Fig. 1A). Farm animal isolates, represented in this study mostly by isolates from poultry and pigs, clustered in clade A distinct from the hospital clade A1 in polyphyletic groups, confirming that there is no distinct clade A2 representing isolates from farm animals (2, 7), in contrast to what was reported previously (6). Pig and poultry isolates were grouped in a limited number of distinct SCs, with 88% of pig isolates grouping in SCs 29 and 30 and 93% of poultry isolates grouping in SCs 24, 25, and 35 (Data Set S1).

### Completed plasmid sequences show extensive modularity.

To elucidate whether plasmids have shaped the observed E. faecium population structure, we first fully resolved the plasmids of E. faecium by performing Oxford Nanopore Technologies sequencing (ONT) and subsequently constructed a hybrid assembly of 62 E. faecium isolates. These isolates were selected to capture the highest plasmidome variability present in our 1,644 clade A E. faecium isolates based on PlasmidSPAdes predictions (15) and a homology search against a curated database of replication initiator proteins in enterococci (11), as previously described (13) (see Text S1).

10.1128/mBio.03284-19.1 TEXT S1 Detailed characterization and description of the results and methods followed in the main manuscript. Download Text S1, DOCX file, 0.1 MB. Copyright © 2020 Arredondo-Alonso et al. 2020 Arredondo-Alonso et al. This content is distributed under the terms of the Creative Commons Attribution 4.0 International license.

Hybrid assemblies resulted in 48 completed (finished) chromosome sequences (and 14 chromosomes distributed among two contigs or more), 305 plasmids, and 6 phage sequences present in single circular contigs (Data Set S1). The 48 complete chromosomes ranged in size from 2.42 to 3.01 Mbp. Hospitalized patient isolates (*n* = 32) had the largest chromosomes (mean, 2.82 Mbp), whereas poultry isolates (*n* = 2) carried the smallest chromosomes (mean, 2.42 Mbp). Notably, hospitalized patient isolates had up to 20% larger chromosomes than E. faecium from other sources, which highlights the considerable genomic flexibility of this organism.

**FIG 2:**
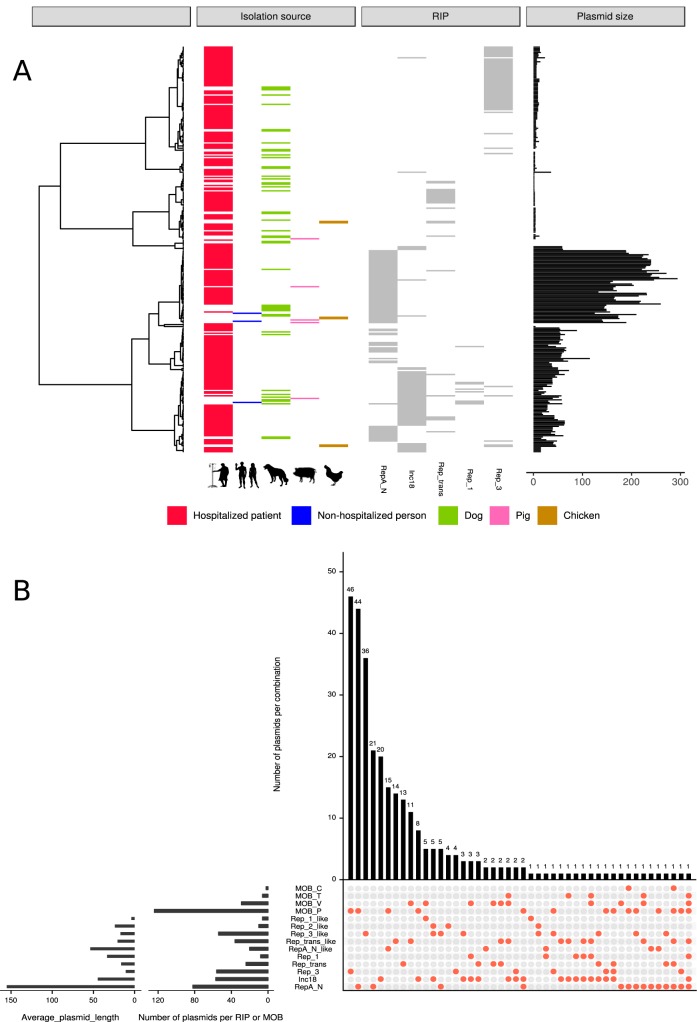
Overview of completed plasmid sequences (*n* = 305). (A) Pairwise Mash distances (k = 21, s = 1,000) of the completed plasmid sequences (*n* = 305) were transformed into a distance matrix and clustered using hierarchical clustering (ward.D2). Node positions in the dendrogram were used to sort and represent in different panels: (i) isolation source, (ii) replication initiator gene (RIP), and (iii) plasmid size (kbp) of the completed plasmid sequences. (B) Intersection plot of the combination of RIP and relaxases found in the set of completed plasmid sequences with associated RIP sequences (*n* = 294).

The set of 305 completed plasmid sequences ranged in length from 1.93 to 293.85 kbp (median, 15.15 kbp; mean, 53.48 kbp) (Fig. 2A, S1, and S2). Hospitalized patient isolates (*n* = 43) with complete plasmid sequences (*n* = 247) contained the highest number of plasmids (mean, 5.70), and their cumulative plasmid length was substantially larger than those from other isolation sources (mean, 308.01 kbp).

10.1128/mBio.03284-19.2 FIG S1 Intersection plot of the combination of RIP and relaxases found in the set of complete plasmid sequences present in long-read sequenced isolates indicating the isolation source in which they were identified. Download FIG S1, EPS file, 0.9 MB. Copyright © 2020 Arredondo-Alonso et al. 2020 Arredondo-Alonso et al. This content is distributed under the terms of the Creative Commons Attribution 4.0 International license.

We characterized these plasmids using a standard classification (11) based on (i) presence of replication initiator proteins (RIP) (Data Set S1) and (ii) presence of relaxases (MOB) (Data Set S1). A considerable proportion of plasmids (48/294 [16%]) were multireplicon plasmids, with plasmids encoding up to four different RIP gene families, indicating a high degree of plasmid modularity (see Fig. S1). This was most prominent in Rep_1 and Inc18 family plasmids, which contained at least one other RIP with a frequency of 1.0 (8/8) and 0.53 (30/57) (Fig. 2B), respectively. The predominant RIP family RepA_N (*n* = 82) was mainly encoded by large plasmids (mean plasmid length, 155.3 kbp) and was less frequently associated with other RIP sequences (*n* = 15, 18%) (Fig. 2B). Plasmids encoding the Rep_3 family (*n* = 56; mean plasmid length, 12.4 kbp) and Rep_trans (*n* = 24; mean plasmid length, 25.7 kbp) were less frequently present in multireplicon plasmids (*n* = 6, 11%) (Fig. 2B). No RIP family was characterized for 11 plasmids (mean plasmid length, 9.6 kbp).

The observed modularity of E. faecium plasmids became even more apparent when relaxase gene families were linked to the fully sequenced plasmids. All identified relaxases cooccurred in plasmids with different RIP genes and even in multireplicon plasmids (Fig. 2B). In total, we observed 46 different Rep-relaxase combinations (Fig. 2B). A more extensive characterization of mosaicism of plasmid sequences is available in Text S1.

### Hospitalized patient isolates have the largest predicted plasmidome sizes.

To predict the plasmidome content present in the other 1,582 E. faecium isolates that were only sequenced with short-read technology, we previously used the information derived from the completed plasmid sequences to develop and validate a machine-learning classifier called mlplasmids (13). The classifier achieved an accuracy of 0.95 and an F1 score (harmonic mean between precision and recall) of 0.92 on a test set of E. faecium sequences generated by short-read sequencing. A more extensive description of the classifier validation and its performance compared to that of existing plasmid prediction tools can be found in the study by Arredondo-Alonso et al. (13).

mlplasmids was used on the present collection of E. faecium isolates, resulting in an average number of base pairs predicted as plasmid derived of 240,324 bp (52 contigs), while the average number of chromosome-derived base pairs was 2,619,359 bp (113 contigs) per isolate. mlplasmids did not predict plasmid-derived contigs in four isolates, including one isolate that was previously described as plasmid-free (64/3, in this study named E2364) (16).

**FIG 3:**
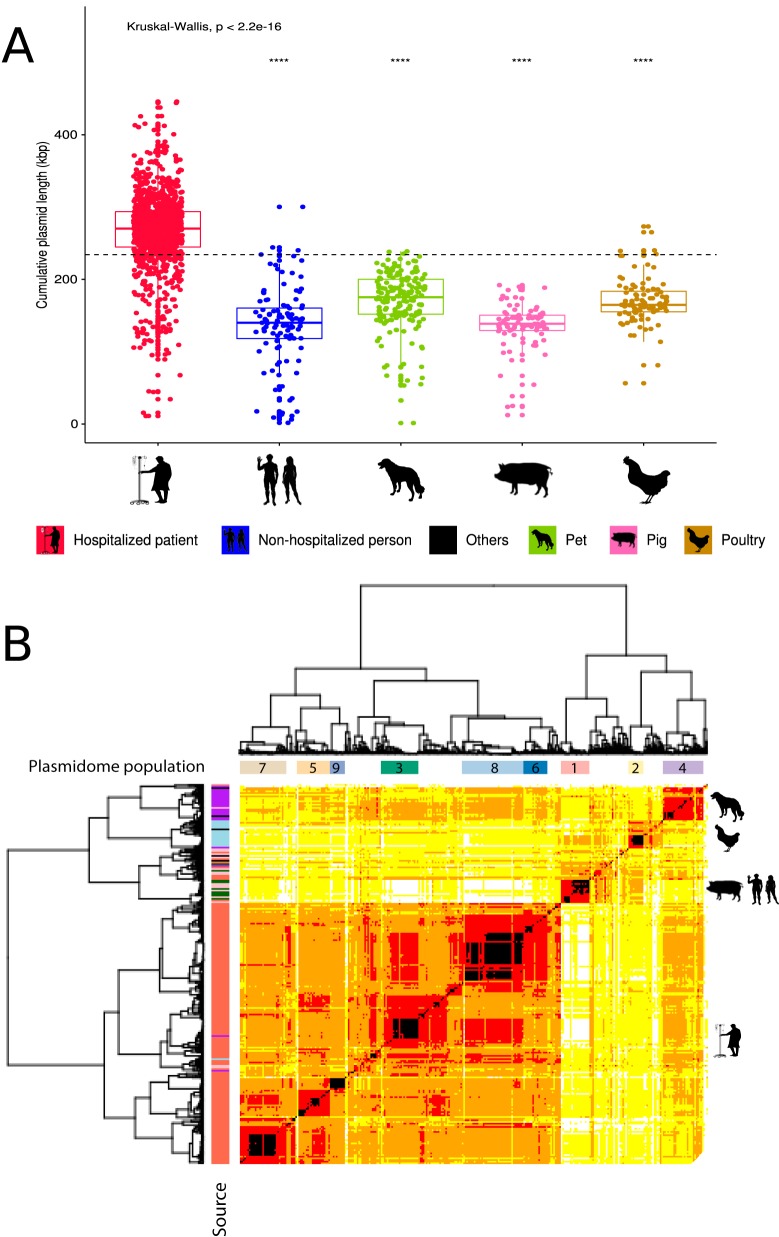
Predicted pan-plasmidome of 1,644 E. faecium isolates. (A) Boxplot of the numbers of base pairs (kbp) predicted as plasmid derived per isolation source. Horizontal dashed line indicates the mean cumulative plasmid length across all the groups. (B) Pairwise Mash distances (k = 21, s = 1,000) of plasmid-predicted contigs in 1,607 isolates were transformed into a distance matrix and clustered using hierarchical clustering (ward.D2). Based on the quantile function of our defined gamma distribution, we grouped isolates in five blocks: black (0 to 0.01), red (0.01 to 0.25), orange (0.25 to 0.5), yellow (0.5 to 0.75), and white (0.75 to 1.0). Dissimilarity matrix of the isolates was visualized as a heat map colored based on the previous blocks. We incorporated the defined plasmid populations (*n* = 9) and isolation source information on top and left dendrograms, respectively.

We observed significant differences in the number of base pairs predicted as plasmid derived depending on the source of the E. faecium isolates (*P* < 0.05) (Fig. 3A). Predicted plasmidome size of isolates from hospitalized patients was considerably larger (mean, 276.16 kbp; *P* < 0.05) than that from other isolation sources (Fig. 3). This finding is in line with previous reports which showed that isolates from clade A1 are enriched for mobile genetic elements (6, 17).

### Plasmidome populations are strongly associated with isolation source.

**FIG 4:**
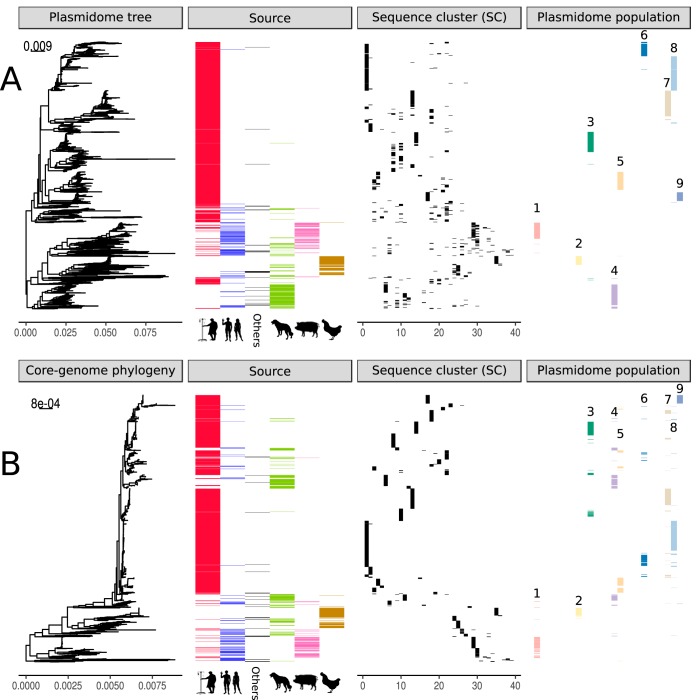
Comparison of reconstructed E. faecium core genome phylogeny and plasmidome trees. The figure includes three different panels: isolation source, sequence cluster (SC), and plasmidome population. (A) bioNJ tree based on the dissimilarity matrix of Mash distances (k = 21, *n* = 1,000) of 1,607 isolates uniquely considering plasmid-predicted contigs. (B) RAxML core genome tree based on 955 E. faecium core genes in 1,644 clade A strains.

To structure the pan-plasmidome of E. faecium, we determined pairwise distances of isolates based on the k-mer content of their predicted plasmidome. We computed a neighbor-joining tree (bioNJ) to cluster E. faecium isolates exclusively on the basis of gain and loss of plasmid sequences (Fig. 4A). During this analysis, 37 isolates were excluded, as they showed no signs of plasmid carriage signatures based on their distribution of pairwise distances (see Fig. S3).

10.1128/mBio.03284-19.3 FIG S2 Boxplot of the distribution in length from the plasmids identified in our set of long-read sequences isolates (*n* = 59) with complete plasmid sequences. Each isolate (*y* axis) was colored based on isolation source (brown, poultry; green, pe; red, hospitalized patient; blue, nonhospitalized person; pink, pig). Isolates are displayed in ascending order based on the total number of plasmids identified. Download FIG S2, EPS file, 0.3 MB. Copyright © 2020 Arredondo-Alonso et al. 2020 Arredondo-Alonso et al. This content is distributed under the terms of the Creative Commons Attribution 4.0 International license.

10.1128/mBio.03284-19.4 FIG S3 Maximizing resolution of the bioNJ plasmid-based tree. (A) bioNJ tree of 1,639 isolates considering plasmid-predicted contigs by mlplasmids. (B). Histogram against fitted density functions of pairwise Mash distances obtained by denscomp function (fitdistrplus R package). Vertical dashed line indicates the Mash distance (0.1224967) used to filter out isolates (*n* = 37) with a higher average pairwise Mash distance. (C) bioNJ plasmid-based tree of 1,607 isolates after exclusion of 32 isolates. Download FIG S3, EPS file, 0.1 MB. Copyright © 2020 Arredondo-Alonso et al. 2020 Arredondo-Alonso et al. This content is distributed under the terms of the Creative Commons Attribution 4.0 International license.

To evaluate the core genome clonality of isolates clustering in the same plasmidome population, we incorporated information regarding isolation source and SCs into the plasmidome tree (Fig. 4A) and core genome phylogeny (Fig. 4B). Isolates with a similar plasmidome contents but different SCs were positioned in different parts of the core genome phylogeny (Fig. 4B), which could be indicative of horizontal transmission of plasmid sequences.

To quantify and formalize these observations of horizontal or vertical transfer of plasmid sequences, we estimated clusters of isolates with similar plasmidomes. The k-mer distances of the plasmidomes were clustered using hierarchical clustering (ward.D2), and we estimated an optimal number of 26 clusters (average silhouette width, 0.45) (Fig. S4A). To enable meaningful statistical inferences, we only considered clusters that contained more than 50 isolates and had an average silhouette width, as a measure of goodness of fit, higher than 0.3 (Fig. S4B). This resulted in 9 clusters that are referred to as plasmidome populations 1 to 9 (Fig. 3B, S4, and S5). We then calculated the SC diversity of all isolates of each plasmidome population (Simpson index) and tested for enrichment of particular isolation sources (Fig. S4B). However, these plasmidome populations may be driven by the k-mer content of large plasmid sequences and could obscure the potential transfer of small plasmid sequences between isolates. An extensive evaluation of the plasmidome populations and potential transfer of the complete plasmid sequences obtained in our study is described in Text S1.

10.1128/mBio.03284-19.5 FIG S4 Definition of plasmidome populations. (A). Average silhouette index for plasmid dissimilarity matrix of pairwise Mash distances clustered using hierarchical clustering (ward.D2), computed for different clustering solutions (2 to 100). We selected 26 as the optimal number of clusters present in the data, which corresponded to an average silhouette index of 0.42. (B) Dendrogram with 26 clusters. From these 26 clusters, we only selected plasmidome populations (*n* = 9) if a particular cluster had a size larger than 50 isolates and an average silhouette index higher than 0.3. Each plasmid population showed overrepresentation of at least one isolation source. Download FIG S4, EPS file, 0.9 MB. Copyright © 2020 Arredondo-Alonso et al. 2020 Arredondo-Alonso et al. This content is distributed under the terms of the Creative Commons Attribution 4.0 International license.

To evaluate the influence of other factors than source category to explain the plasmidome clustering, we modeled the observed plasmid k-mer distances using three linear regression models with three different covariates: source, isolation time, and geographical distance between pairs of isolates. We observed that modeling k-mer distances using exclusively source explained 39% of the variance present in the plasmid k-mer distances, whereas using time (difference in years between the isolates) as covariate explained 29% of the variance. Geographical distance between isolates explained less than 1% of the variance. Finally, we incorporated the three predictors into a multiple linear regression model, which increased the variance explained up to 43%. This elucidated that isolation source was the most important predictor to explain plasmidome clustering, but a difference in time between strains must be considered: isolates which are circulating during the same period of time are more likely to share plasmid sequences. Geographical distance between isolates seems not relevant to explain the observed clustering, which suggests a high mobility and spread of E. faecium plasmid sequences.

### Restriction modification systems, but not CRISPR-Cas, could act as barriers of horizontal gene transfer.

The absence of CRISPR-Cas systems in clade A1 isolates was previously postulated as a plausible explanation for a nondiscriminatory accumulation of plasmid sequences in clade A1 isolates (6, 18). However, we only observed a CRISPR-Cas system in a single non-clade A1 isolate and no occurrence of the recently described Jet system in any of the isolates (19). The absence of a CRISPR-Cas system is therefore unlikely to result in a higher and different plasmidome content of clade A1 isolates from hospitalized patients.

Recently, a novel defense mechanism consisting of a restriction modification (RM) system was postulated as contributing to the subspeciation of E. faecium (20). The specificity of the RM system resides in the S subunit, which binds to different DNA sequences by two target recognition domains. In our collection, we also identified the S subunit (WP_002287733) as present and enriched in clade A1 isolates (*P* < 0.05), whereas the subunits M and R were identical in both clade and non-clade A1 isolates and always present together with the S subunit. Furthermore, we identified 8 novel S subunit variants in our set of 62 isolates with complete genome sequences. Of these, four variants (E1774_00555, E7313_02981, E4413_00571, and E4438_00276) were significantly enriched in clade A1, while two other variants (E0139_00520 and E4227_02943) were enriched in non-clade A1 isolates, which reinforces the hypothesis that different RM systems contribute to the differentiation of the plasmidome content between isolation sources (Text S1).

### Characterization of genes driving the plasmidome populations.

To identify which genes were potentially driving the observed plasmidome populations (*n* = 9), we determined, for each plasmidome population, which genes were present in more than 95% of the isolates and defined those as plasmidome population core genes. We further characterized these genes using eggNOG to retrieve the cluster of orthologous genes (COG) and associated KEGG pathways. These plasmidome population core genes were then searched in our set of complete plasmid sequences to identify the type of replicon sequences bearing these genes, such as large RepA_N or Inc18 plasmids.

Most of the plasmidome population core genes belonged to COG S (unknown function) and COG L (DNA replication, recombination, and repair) (Fig. S6; Data Set S1). Within these two COG groups, we identified functions such as toxin-antitoxin (TA) systems, involved in the stabilization of large plasmid sequences (e.g., RelE/AbrB, MazEF, and HicAC systems), and a type IV TA “innate immunity” bacterial abortive infection (Abi) system that protects bacteria from the spread of a phage infection (AbiEi/AbiEii). This TA system interferes with phage RNA synthesis, enables stabilization of mobile genetic elements (21), and was extensively described in lactococcal plasmids (22).

10.1128/mBio.03284-19.6 FIG S5 Clustering quality and diversity of the defined plasmidome populations (*n* = 9). (A) Average silhouette index of each plasmidome population. Size of the point indicates the number of isolates belonging to that particular population. Horizontal dashed line indicates the average silhouette index of the selected clustering solution (k = 26, average silhouette index, 0.42). (B) Simpson indexes and their associated confidence intervals (95%, 1,000 bootstrap replications), based on SC diversity. Download FIG S5, EPS file, 0.1 MB. Copyright © 2020 Arredondo-Alonso et al. 2020 Arredondo-Alonso et al. This content is distributed under the terms of the Creative Commons Attribution 4.0 International license.

10.1128/mBio.03284-19.7 FIG S6 Number of core plasmidome genes (*y* axis) grouped into COG categories (*x* axis) from each plasmidome population (*n* = 9). Download FIG S6, EPS file, 0.2 MB. Copyright © 2020 Arredondo-Alonso et al. 2020 Arredondo-Alonso et al. This content is distributed under the terms of the Creative Commons Attribution 4.0 International license.

Interestingly, we identified some plasmidome population core genes only present in particular populations. For plasmidome population 1 (pig and nonhospitalized isolates), we identified a copper resistance operon (*tcrYAZB*) that provides a mechanism to tolerate high concentrations of this heavy metal as plasmidome population core genes. Copper was commonly used as a growth-promoting agent for pigs (23). However, high levels of copper result in toxicity for the bacterial cells. The *tcrYAZB* operon provides a plasmid survival mechanism to tolerate high concentrations of this heavy metal. In addition, we identified the glycopeptide resistance-encoding *vanA* gene cluster as a plasmidome population core in the population. These genes were harbored on a RepA_N conjugative plasmid of 140 kbp (LR132068.1 and LR135180.1) and colocalized with genes encoding plasmid stabilization systems (RelE/AbrB and AbiEi/AbiEii), which may explain the persistence of this large plasmid in the population.

Plasmidome population 2 (poultry associated) also showed plasmidome population core genes which were exclusively present as core in this population. This included the bile salt hydrolase (BSH) choloylglycine hydrolase and putatively a tetronasin resistance-encoding permease gene. BSH is involved in the deconjugation (hydrolysis) of bile acids, which have antimicrobial activity, especially against Gram-positive bacteria (24). Therefore, acquisition of BSH could serve as a selective advantage for E. faecium for gut colonization. In a recent review, BSHs have been described as the gatekeepers of bile acid metabolism and host-microbe cross talk in the gastrointestinal tract (25). In addition, as mentioned, homologous searches revealed only hits for E. faecium strains isolated from chicken, but we also obtained hits for Enterococcus cecorum (100% similarity in amino acids [AA]), which is a species mainly found in birds. In both strains, BSH was located downstream of the same site-specific recombinase, which highly suggests HGT between these 2 species. We also observed a tetronasin resistance gene as a plasmidome population core gene. The presence of this gene on a mobile element among E. faecium poultry isolates was previously described and may be the result of selective pressure due to the wide use of ionophores, e.g., tetronasin for coccidiosis prophylaxis in poultry (26). Interestingly, this gene is often colocated on a plasmid with Tn*1546* encoding vancomycin resistance and TA systems.

In the case of the hospital-associated plasmidome populations (3, 5, 6, 7, 8, and 9), we characterized some genes present in all these populations. Of these, a locus of three genes putatively encodes an ABC transport system, while one gene encodes an ATP-binding protein and the other two genes encode permeases. These genes were assigned to COG V (defense mechanisms) and were similar to the previously described *vex* locus of Streptococcus pneumoniae. In S. pneumoniae, this gene cluster was initially linked to vancomycin tolerance (27), but Moscoso and coauthors disproved these results (27, 28). Protein analysis of the ATP binding protein Vex2 revealed the presence of domains with similarity to lipoprotein/bacteriocin/macrolide export systems, which may suggest that this system is involved in antibiotic resistance. We also observed antimicrobial resistance genes such as aminoglycoside resistance (*aacA-aphD*) and erythromycin resistance (*erm*) present in the plasmidome population core of all the hospitalized patient populations.

In line with the hypothesis of different routes of hospital adaptation, we observed some plasmidome population core genes that are only present as core in some plasmidome populations associated with hospitalized patients. We observed the presence of a bacteriocin with homology to BacA in populations 5, 7, and 8 and previously described as a plasmid-borne bacteriocin in E. faecalis (29). BacA can act as a more evolved toxin-antitoxin system in which not only daughter cells but also cells from the same generation not bearing the BacA plasmid are excluded. Furthermore, it was demonstrated that plasmid dissemination was more prominent under conditions of fluctuations in the population of E. faecium, since BacA activity exclusively affects dividing cells (29). We also observed a complete phosphotransferase system putatively involved in mannose/fructose/sorbose utilization present in the plasmidome cores of populations 6 and 7. This may provide novel pathways for the utilization of complex carbohydrates in these hospital-associated populations.

A complete characterization of the plasmidome population core genes and the complete plasmid sequences in which these genes are located can be found in Text S1.

### Plasmidome content is the major genomic component driving niche specificity.

To assess which of the genomic components (chromosome or plasmidome) contributed most to source specificity, we compared the distributions of k-mer pairwise distances using three different inputs: (i) whole-genome contigs, (ii) chromosome-derived contigs, and (iii) plasmid-derived contigs. We hypothesized, for source-specific components, that k-mer distances between pairs of isolates belonging to the same source were lower than pairs of isolates from different or random sources. This difference can reflect the association strength between niche and genomic component (whole-genome, chromosome-derived, and plasmid-derived contigs). We followed a bootstrap approach to compare and average k-mer pairwise distances of (i) pairs of isolates from the same isolation source (within-source group), (ii) pairs of isolates belonging to different isolation sources (between-source group), and (iii) pairs of isolates randomly selected (random group).

**FIG 5:**
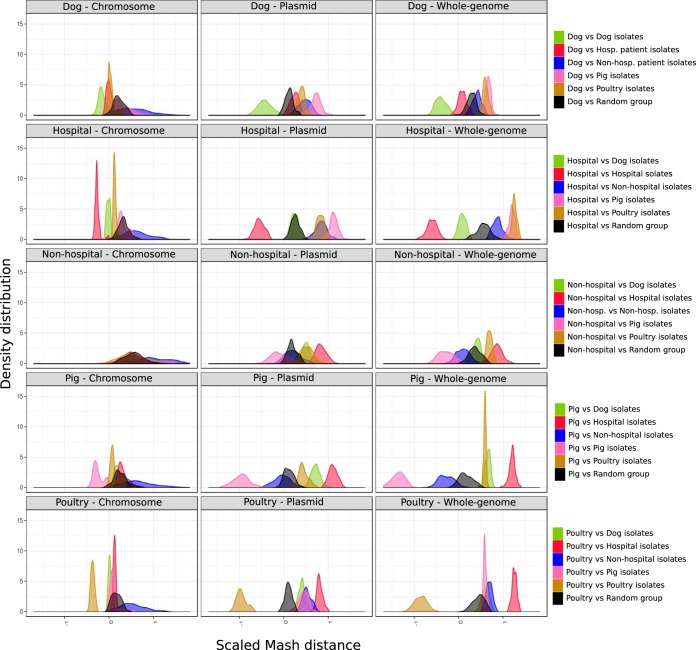
Evaluation of the source specificity from each genomic component. Mash distances computed from chromosome-predicted (first column), plasmid-predicted (second column), and whole-genome (third column) contigs were scaled and compared between all the isolation sources. Each row corresponds to a particular isolation source (e.g., first row refers to dog isolates) and the distribution of pairwise distances against other sources (dog in green, hospitalized patient in red, nonhospitalized person in blue, pig in pink, poultry in brown, and random isolates in black) for each genomic component. These average distances were computed using a bootstrap approach (100 iterations). The distribution of pairs of isolates from the same source type with respect to the distribution of pairs from random isolates (black group) reflects the specificity of the genome component in each source. If pairs from the same source deviate to the left, it indicates a higher specificity of that particular genomic component, whereas a deviation to the right with respect to the pairs of random isolates (black group) indicates a lower specificity than expected by chance.

Whole-genome contigs explained most of the source specificity of all the isolation sources except for nonhospitalized person isolates, based on the highest k-mer pairwise distance differences between isolates from the same source (within source) and randomly selected isolates (Fig. 5 and S7).

However, with the exception of nonhospitalized person isolates, the plasmidome contribution was higher than the chromosome contribution to explain source specificity. This was based on the highest difference in k-mer pairwise distances between isolates from the same (within-source group) and different (between-source group) sources when comparing the plasmidome versus the chromosome (Fig. 5 and S7).

10.1128/mBio.03284-19.8 FIG S7 Differences in the observed means of average pairwise distances when comparing within-host and between-host groups against our defined random group of isolates. Each line corresponds to a different genomic component (whole genome, black solid; chromosome, dotted red; plasmid, dashed purple), and test significance is indicated based on shape (triangle, nonsignificant; circle, significant). Download FIG S7, EPS file, 0.3 MB. Copyright © 2020 Arredondo-Alonso et al. 2020 Arredondo-Alonso et al. This content is distributed under the terms of the Creative Commons Attribution 4.0 International license.

Most notably, we observed significant similarities of the whole genome and chromosome of dog and hospitalized patient isolates (positive difference, 0.20; *P* < 0.05) but a significant dissimilarity between these two sources when considering their plasmidomes (negative difference, −0.13; *P* < 0.05) (Fig. 5 and S7). In addition, pig and nonhospitalized person isolates had significantly similar plasmidomes as observed by a small difference in k-mer distances (positive difference, 0.15; *P* < 0.05), corroborating the postulated exchange of plasmid sequences between these two groups (Fig. S7).

## DISCUSSION

We used a combination of ONT long-read and Illumina short-read technologies to perform a comprehensive analysis of the pan-plasmidome of the nosocomial pathogen E. faecium which has evolved in different niches. The high number of multireplicon plasmids consisting of several combinations of RIP families confirmed the high levels of mosaicism previously observed for E. faecium plasmids, which challenges the classification of *Enterococcal* plasmids based on RIP schemes (30).

We observed that the total plasmidome size of isolates from hospitalized patients was substantially larger than that from animal isolates and isolates from nonhospitalized persons. Moreover, clustering of k-mer pairwise distances from our set of predicted plasmid sequences revealed a high level of diversity in E. faecium plasmidomes. We estimated the potential contribution of different genomic components (whole genome, chromosome, and plasmid) to source specificity and observed that the plasmidome explains source specificity in dogs and hospitalized patients, while their corresponding core genomes share an evolutionary history. This finding suggests that either the hospital-adapted population was founded by a host jump from the canine population or, alternatively, the host jump happened in the other direction. In line with previous reports (31, 32), we observed that nonhospitalized person isolates in our collection shared their plasmidomes with pig isolates, which indicates an exchange of plasmids or strains between both sources.

Source specificity of plasmid sequences was highest in pigs and poultry isolates and significantly differed from the other sources, but also, the plasmidomes of clinical isolates were highly dissimilar to isolates from other sources. This suggests that the pan-plasmidome of E. faecium plays a role in the emergence of this organism as a nosocomial pathogen of major importance. There was not, however, a single preferred plasmidome configuration for hospital patient isolates, but rather, these isolates were associated with six different plasmidome populations, indicating different possible routes of plasmid acquisition within the hospital environment.

The existence of distinct host-associated plasmidome populations indicates that the dissemination of plasmids within the E. faecium population is restricted. The presence of particular S subunit variants belonging to a type I RM system enriched either in clade A1 isolates or non-clade A1 isolates in the E. faecium population suggests that they play an active role as HGT barriers between isolates from different sources (20). Restriction modification systems potentially limit the exchange of plasmid sequences and might contribute to source specificity. In a few cases, we observed the presence of single isolates from a specific source in plasmidome populations dominated by a different source, as exemplified in the case of plasmidome population 4 (dog enriched) and the hospitalized patient isolate E8172. In this case, we identified a similar RepA_N conjugative plasmid potentially transmitted from or to dogs to or from that particular hospitalized patient’s isolate. The presence of identical S subunit variants between hospitalized patient and dog isolates (clade A1 enriched) could enable an occasional exchange of plasmid sequences between different sources.

Exploration of the core genes of the predicted plasmidome populations revealed that most plasmid genes are poorly characterized. We further characterized some of the plasmid genes with an unknown function as toxin-antitoxin systems. The widespread occurrence of these selfish systems is indicative of their importance in plasmid maintenance and stabilization. Previous reports have shown a high prevalence of particular toxin-antitoxin systems, such as *mazEF*, in E. faecium clinical isolates (33). They could contribute to the stabilization of plasmid-mediated antibiotic resistance by the maintenance of a single plasmid structure and might thus provide an interesting alternative target for antibiotic therapy.

We also identified a set of copper resistance genes (*tcrYAZB* operon) in the core plasmidome of population 1 (pig and nonhospitalized associated). Copper was used as a growth-promoting agent in piglets (34), and high levels of copper are toxic for most bacterial species. The acquisition of copper resistance genes may have contributed to the adaptation of E. faecium to environmental constraints imposed by pig farming. Recently, Gouliouris et al. also described the same copper resistance operon as overrepresented in pig isolates, thus confirming that this set of plasmid-borne genes has played an important role in E. faecium survival in farms (35). Those plasmid genes were identified in our set of complete plasmid sequences and were present in a RepA_N conjugative plasmid (140 kbp) identified in pig and nonhospitalized isolates. Furthermore, we identified a BSH gene widely present in the poultry-associated plasmidome population. E. faecium was previously characterized as one of the microorganisms with the highest level of BSH activity in the intestines of chickens (36) and capable of developing new mechanisms to tolerate a high concentration of bile salts (37). The BSH gene described here could be functionally responsible for the bile tolerance of poultry isolates.

The presence of several plasmid genes involved in carbohydrate metabolism and utilization in plasmidome populations associated with hospitalized patients may indicate the acquisition of novel pathways to process complex carbohydrates. This observation is in line with previous reports (6, 38) in which phosphotransferase systems enriched in clade A1 isolates and encoded by mobile genetic elements were fundamental for E. faecium during gastrointestinal (GI) tract colonization. The high frequency of plasmid genes with an unknown function or corresponding to hypothetical proteins could mask the presence of other plasmid-mediated mechanisms contributing to niche adaptation. This highlights the importance of further functional studies to elucidate the roles of these plasmid genes.

The observations that plasmid sequences are highly informative for source specificity and that particular genes may have a clear benefit for E. faecium in particular niches suggest that the distribution of plasmid genes among E. faecium isolates is regulated by complex ecological constraints, and thus contributes to niche adaptation, rather than by opportunities arising from physical interactions between different sources. Of note, this approach does not calculate the contribution of a single genomic sequence but of the whole genomic component (plasmid or chromosome) to the niche specificity. Small chromosomal alterations or rearrangements could also be involved and play an important role in niche specificity.

Based on our findings, we elucidated that isolation source was the most important predictor to explain the observed plasmidome clustering and indicated that isolates from the same niche can exchange plasmid sequences during the same time frame. Combining extensive short- and long-read sequencing of a large collection of isolates from a diverse set of sources, as reported here for E. faecium, may serve as a broadly applicable approach to study the pan-plasmidome of evolutionary and ecologically diverse populations.

## MATERIALS AND METHODS

### Genomic DNA sequencing, assembly, and characterization of plasmids.

Detailed description of Illumina and ONT sequencing is available in Text S1 in the supplemental material and in the study by Arredondo-Alonso et al. (13), which includes a full description of ONT selection of E. faecium isolates (*n* = 62) and consecutive hybrid assembly using Unicycler (39). Characterization of fully assembled plasmids is also described in Text S1.

### Population genomic analysis.

Pangenomes for the entire genome data set (1,684 strains) and the clade A data set (1,644 strains) were created using Roary (40) with default settings. A core gene alignment was generated using the –mafft option in Roary, resulting in a core gene alignment of 859 genes for the entire data set and of 978 genes for the clade A data set. To estimate recombination events and to remove them from the core genome alignment, we used BratNextGen with default settings, including 20 hidden Markov model (HMM) iterations, 100 permutations run in parallel on a cluster, and 5% significance level, similar to those in earlier publications (41, 42). To determine sequence clusters (SCs) in the core genome alignment where significant recombinations had been removed, we used 5 estimation runs of the hierBAPS method (43) with 3 levels of hierarchy and the prior upper bound for the number of clusters ranging in the interval 50 to 200. All runs converged to the same estimate of the posterior mode clustering. We considered the second level of hierarchy (postBNGBAPS.2) to determine SCs in our collection. To estimate a phylogenetic tree, we used RAxML (44) with the GTR+Gamma model on a core gene alignment stripped of recombination. The bootstrap option was disabled in RAxML due to an extremely long runtime.

### CRISPR-Cas and restriction modification system detection.

To detect CRISPR-Cas arrays present in our set of 1,644 E. faecium isolates, we first used CRISPRDetect (version 2.2) (45), and detected hits were further validated using CRISPRCasFinder (version 1.1.1) (46).

To observe the presence of the restriction modification system described by Huo et al. (20), we retrieved the nucleotide sequences of the S subunit (WP_002287733.1), M subunit (WP_002287732.1), and R subunit (WP_002287735.1) from the E. faecium genome sequence (NZ_GG688488). We screened for the presence of these subunits in our entire collection of isolates (1,644) using Abricate and defined a 95% minimum identity and 90% coverage as thresholds (version 0.8.2). Later, we focused our analysis on the set of complete genome isolates (62) and performed a multiple-sequence alignment on the protein level of all the S subunits identified using Clustal Omega (version 1.2.4) (47). Based on the multiple-sequence alignment, we defined 8 novel S subunit variants that were tested for enrichment in either clade A1 or non-clade A1 isolates using a Fisher exact test with the function fisher.test from R stats package (version 3.4.4).

### Predicting the plasmidome content of short-read sequenced E. faecium isolates.

To determine the plasmidome content of the remaining 1,582 isolates, we used mlplasmids (13). mlplasmids (version 1.0.0) was run, specifying “Enterococcus faecium” model and a minimum contig length of 1,000 bp. For further analysis, we discarded predicted contigs with a posterior probability lower than 0.7 of belonging to the assigned class (chromosome/plasmid; https://gitlab.com/sirarredondo/efaecium_population/raw/master/Files/mlplasmids_prediction/prediction_svm.tsv). Differences in the numbers of chromosome- and plasmid-derived base pairs predicted by mlplasmids between hospitalized patient isolates and other isolation sources were assessed using the Kruskal-Wallis test (significance threshold, 0.05) available in ggpubr package (version 0.1.7) (48).

We calculated pairwise Mash distances (k = 21, s = 1,000; version 1.1) between isolates (*n* = 1,640), only considering plasmid-predicted contigs. We reconstructed a plasmidome tree with the bioNJ algorithm implemented in the R ape package (version 5.1) using computed Mash distances (49, 50). The resulting phylogenetic tree was midrooted using the midpoint function in the R phangorn package (version 2.4.0) (51). To improve the resolution of the bioNJ tree, we observed the distribution of the computed Mash distances and fitted a gamma distribution using the fitdist function (distr = “gamma” and method = “mle”) available in the R fitdistrplus package (52). We discarded isolates with an average pairwise mash distance superior to 0.12, which was calculated using the qgamma function (*P* = 0.9, shape = 2.344073, rate = 35.870449, lower.tail = TRUE) in the R stats package (version 3.4.4). All remaining isolates (*n* = 1,607) were used to reconstruct the plasmidome tree.

We used the function NbClust (method = “ward.D2” and index = “silhouette”) available in the R NbClust package (version 3.0) (53) to evaluate an optimal number of clusters derived from pairwise Mash distances. We computed hierarchical clustering using the hcut function (method = “ward.D2”, isdiss = TRUE, k = 26) and cut the resulting dendrogram specifying 26 clusters. For each resulting cluster, we uniquely defined plasmidome populations (*n* = 9) based on two criteria: (i) clusters with more than 50 isolates and (ii) an average silhouette width greater than 0.3.

Correlation of plasmidome populations and isolation sources was determined using a one-sided Fisher exact test (alternative = “greater”) from the fisher.test function (R stats package version 3.4.4) and naive *P* values were adjusted using the Benjamini-Hochberg (BH) method implemented in p.adjust function (R stats package, version 3.4.4). We considered an adjusted *P* value threshold of 0.05 to determine enrichment of isolation sources for specific plasmidome populations. We incorporated metadata and plasmid population information into plasmid bioNJ and the E. faecium core genome tree using the R ggtree package (version 1.13.3). Simpson index based on SC diversity (postBNGBAPS.2 group) (Data Set S1) and its associated 95% confidence interval from 1,000 bootstrap replications was computed using the R package iNEXT (version 2.0.19) (54).

We evaluated the influence of two other covariate (time and distance) in the clustering derived from Mash distances. For each pair of isolates, we determined (i) if they belonged to the same or different isolation source, (ii) time difference (in years) between their isolation times, and (iii) geographical distance. To calculate the geographical distance, we considered the latitude and longitude of each isolate and used the distm function (R geosphere package, version 1.5-7). We fitted three linear regression models (function lm in R stats package, version 3.4.4) considering as response the pairwise Mash distances and the previous defined covariates. For each model, we retrieved its adjusted *R*^2^ to explain the percentage of variance explained by each covariate. We combined all three covariates in a multiple linear regression model using the function lm (R stats package, version 3.4.4) and further evaluated the observed correlations by performing a permutation test with the function lmp from the package lmPerm (version 2.1.0) (55).

### Contribution of genomic components to source specificity.

To evaluate the contribution of genomic components on source specificity, we considered three different inputs: (i) Mash pairwise distances from whole-genome contigs, (ii) Mash pairwise distances from chromosome-derived contigs, and (iii) Mash pairwise distances from plasmid-derived contigs. Pairwise distances were scaled using the scale function (scale = TRUE, center = TRUE) from the R stats package (version 3.4.4). For each isolation source (hospitalized patient, dog, poultry, pig, and nonhospitalized person), we used a bootstrap approach (100 iterations) to calculate the average pairwise distances of 50 random isolates belonging to the following combinations: (i) pairs of isolates belonging to the same niche (within-source group), (ii) pairs of isolates belonging to different niches (between-source group), and (iii) pairs of isolates belonging to random isolation sources (random group). This random group consisted of an artificial group in which we merged 50 random isolates belonging to any of the five isolation sources after sampling 100 isolates from each of the sources to avoid overrepresentation of hospitalized patient isolates. This random group was used to statistically assess whether the distribution of pairwise distances belonging to within-source and between-source groups differed from that of random pairwise distances. We used a one-way analysis of variance (ANOVA) test (aov function, R stats package version 3.4.4) and computed differences in the observed means using Tukey’s honestly significant difference (HSD) function available in the R stats package (version 3.4.4). Significant (adjusted *P* < 0.05) positive and negative observed differences of the means were considered indications of niche adaptation similarity and dissimilarity, respectively.

### Estimating the core plasmidome of the defined populations.

We used Roary (version 3.8) (40) to define orthologous groups present in each plasmidome population by defining a threshold of 95% amino-acid-level similarity and nonsplitting paralogues. We defined the core plasmidome of each population as the total number of core genes (OGs present in more than 99% isolates) and soft-core genes (OGs present in more than 95% of the isolates but less than 99% of the isolates). To group these core plasmidome genes into different COG categories, we used eggNOG (version 1.0.3-5-g6972f60) with the translate option and the bacterial database (4.5.1) provided.

### Data availability.

The complete code used to generate the analysis reported in the manuscript is publicly available at the following GitLab repository: https://gitlab.com/sirarredondo/efaecium_population.

Illumina NextSeq 500/MiSeq reads of the 1,644 E. faecium isolates used in this study have been deposited in the following European Nucleotide Archive (ENA) public project: PRJEB28495. Oxford Nanopore Technologies MinION reads used to complete the 62 E. faecium genomes are available under the following figshare projects: 10.6084/m9.figshare.7046804 and 10.6084/m9.figshare.7047686.

Hybrid assemblies generated by Unicycler (v.0.4.1) are available under the ENA and NCBI project PRJEB28495 and also retrievable at the following GitLab repository: https://gitlab.com/sirarredondo/efaecium_population/tree/master/Files/Unicycler_assemblies. Annotation of the complete genome sequences generated in this study are available on NCBI under BioProject PRJEB28495.

Pangenomes of the observed plasmidome populations and eggNOG annotation are available at https://gitlab.com/sirarredondo/efaecium_population/tree/master/Files/Plasmid_populations.

Exploratory analysis of our data and metadata set is available at the following microreact project: https://microreact.org/project/BJKGTJPTQ.
