# Supplementary material for: Plasmids Shaped the Recent Emergence of the Major Nosocomial Pathogen Enterococcus faecium
Source: mBio. 2020 Feb 11;11(1):e03284-19. doi: 10.1128/mBio.03284-19 (PMC7018651; doi:10.1128/mBio.03284-19)
Supplement: TEXT S1 [file mBio.03284-19-s0001.docx]

Supplementary Text S1: Plasmids shaped the recent emergence of the major nosocomial pathogen *Enterococcus faecium*

Arredondo-Alonso S^a^, Top J^a^, McNally A^b^, Puranen S^c,d^, Pesonen M^c,d^, Pensar J^d^, Marttinen P^d^, Braat JC^a^, Rogers MRC^a^, van Schaik W^b^, Kaski S^c^, Willems RJL,^a$^#, Corander J^d,e,f$^#, Schürch AC^a^#

^a^ Department of Medical Microbiology, University Medical Center Utrecht, Utrecht, The Netherlands

^b^ Institute of Microbiology and Infection, University of Birmingham, Birmingham, United Kingdom

^c^ Department of Computer Science, Aalto University, FI-00076 Espoo, Finland

^d^ Department of Mathematics and Statistics, Helsinki Institute of Information Technology (HIIT), FI-00014 University of Helsinki, Finland

^e^ Pathogen Genomics, Wellcome Trust Sanger Institute, Cambridge CB10 1SA, UK

^f​^ Department of Biostatistics, University of Oslo, 0317 Oslo, Norway

SA, JT contributed equally to this article

ACS, JC and RJLW contributed equally to this article

#Address correspondence to Schürch AC, [a.c.schurch@umcutrecht.nl](mailto:a.c.schurch@umcutrecht.nl), Corander J, [jukka.corander@medisin.uio.no](mailto:jukka.corander@medisin.uio.no), Willems RJL, [rwillems@umcutrecht.nl](mailto:rwillems@umcutrecht.nl)

Running title: Plasmids of the nosocomial pathogen *E. faecium*

# Supplementary Text (Results)

# **Suppl. Results: Characterization of completed plasmid sequences obtained by ONT sequencing**

The completely sequenced plasmids were first characterized based on their replication initiator proteins (RIP). Most of the completed plasmid sequences contained a RepA_N initiator sequence (n = 82) with similarity versus RIP sequences described in pLG1 megaplasmid (n = 55, accession number ADO66907) and the non-conjugative pRUM plasmid (n = 27, accession number NP_863172). RepA_N family was found in large plasmids (mean = 155.2 kbp) (Figure 2B), occasionally associated with other RIP sequences (n = 15, freq = 0.18) (Figure 2B) and present in hospitalized patients (n = 63), dog (n = 12), pig (n = 3), non-hospitalized persons (n = 2) and chicken isolates (n = 2) (Suppl. Figure S1). We also identified plasmids containing RepA_N-like (<80% identity) initiators (n = 20) in medium plasmids (mean 53.9 kbp).

Also the Inc18 family was ubiquitous in the collection of plasmid sequences (n = 57) and present in plasmids with a medium size (mean = 44.7 kbp) (Figure 2B). Plasmids bearing Inc18 family showed higher levels of mosaicism than RepA_N plasmids and were frequently present in multireplicon plasmids (n = 30 ; freq = 0.53). In this study, we mainly found Inc18 sequences with similarity to the initiator sequence from pRE25 plasmid (n = 44, accession number Q9AL28) which was originally identified in *E. faecalis* from a raw-fermented sausage and associated with multiple antibiotic resistance genes [(1)](https://paperpile.com/c/BW0bbP/e0vxc). We identified Inc18 plasmids in isolates from hospitalized patients (n = 49), dog (n = 7), chicken (n = 2), pig (n = 1) and non-hospitalized persons (n = 1) (Suppl. Figure S1).

The Rep_3 family was mostly found on small plasmids (n = 56, mean plasmid length = 10.8 kbp) (Figure 2B) and rarely present in multireplicon plasmids (n = 6, freq = 0.11.) (Figure 2B). We found Rep_3 sequences with similarity to other small theta-replicating plasmids such as *Enterococcus durans* pGL (n = 22, accession number ADW93773) or *E. faecium* p200B (n = 8, accession number BAF44066). In contrast to RepA_N and Inc18 families, we only found Rep_3 sequences in isolates from hospitalized patients (n = 49), dogs (n = 5) and chickens (n = 2). We detected a high number of plasmids containing Rep_3-like (<80% identity) initiator sequences (n = 54, mean pl. length = 17.1 kbp) and were occasionally associated with other rip families (n = 19, freq = 0.35).

Rep_trans family (n = 24) was mainly identified in plasmids with a medium size (mean = 16.36 kbp) (Figure 2B) and occasionally present in multireplicon plasmids (n = 9, freq = 0.38) (Figure 2B). We mainly found similarity with Rep_trans sequence from pRI1 (n = 16, accession number YP_001672021), a small cryptic mobilizable *E. faecium* plasmid from human and animal origin [(2)](https://paperpile.com/c/BW0bbP/p3TIJ). Plasmids bearing Rep_trans family were present in hospitalized patients (n = 19), dog (n = 3) and chicken (n = 2) isolates (Suppl. Figure S1). We also characterised Rep_trans-like (<80% identity) sequences (n = 36, mean pl. length = 20.8 kbp) with a similar frequency of being associated to other rip sequences (n = 11, freq = 0.31).

Rep_2-like (<80% identity) sequences (n = 10) were present in small-medium plasmids (24.0 kbp), frequently present in multireplicon plasmids (n = 6, freq = 0.6), similar to pJB01 (n = 9, accession number YP_138502) [(3)](https://paperpile.com/c/BW0bbP/LeyPh) and only present in hospitalized (n = 8) and dog isolates (n = 2) (Suppl. Figure S1).

Finally, we described another known *Enterococcus* Rip family corresponding to Rep_1 (n = 8, mean = 33.4 kbp). This Rip group was only present in multireplicon plasmids in association with RepA_N and Inc18 families (n = 8, freq = 1.0). All Rep_1 sequences had similarity to a previously described non-functional Rep of the non-conjugative pAMα1 (accession number NP_863351) plasmid from *E. faecalis* [*(4)*](https://paperpile.com/c/BW0bbP/sl70P). Our findings were in accordance with previous reports suggesting Rep_1 family may not be functional and other rip initiators are required for plasmid replication [(5)](https://paperpile.com/c/BW0bbP/o19PW). Plasmids carrying Rep_1 initiators were found in patients (n = 5), dogs (n = 2) and non-hospitalized person isolates (n = 1). We additionally found Rep_1-like (<80% identity) sequences (n = 6) present in small plasmids (mean = 4.0 kbp), not associated with other replication initiator families and with similarity to Rep_1 sequences from *E. faecalis* pTEF1 (n = 6, accession number NP_816941) and *E. faecium* pNJAKD (n = 2, accession number YP_004747351).

MOB_P family was the most predominant relaxase family (n = 124) (Figure 2B) and present in plasmids with a single RepA_N (n = 44) or Rep_3 (n = 46) initiator sequence (Figure 2A). This relaxase family was also found in RepA_N-like (n = 15), Inc18 (n = 8), and other multireplicon plasmids (n = 11). MOB_V family was mainly found in plasmids carrying a single Rep_trans-like (n = 11), Rep_1-like (n = 5), Rep_trans (n = 2) families and multireplicon plasmids (n = 11) containing different combinations of Inc18, Rep_1, RepA_N and Rep_1 families (Figure 2A). MOB_T family was identified in multireplicon plasmids containing a Rep_trans-like group and several combinations of Inc18, RepA_N an Rep_1 sequences (n = 6). MOB_C was only found in RepA_N plasmids (n = 2) including a multireplicon plasmid (RepA_N, Rep_trans and Rep_trans-like) (Figure 2A).

**Suppl. Results: Restriction-modification systems, but not CRISPR-cas, could act as barriers of horizontal gene transfer**

Recently, a new type I restriction modification (RM) system has been described in *E. faecium* [*(6)*](https://paperpile.com/c/BW0bbP/71OAF). This RM system is composed of three subunits (M, R, and S). The latter subunit is responsible for the recognition and binding to foreign DNA sequences through the presence of two target recognition domains and was enriched in a set of clade A1 isolates [(6)](https://paperpile.com/c/BW0bbP/71OAF). A different set of RM systems could act as barrier in the gene exchange of *E. faecium* and contribute to the subspecies separation [(6)](https://paperpile.com/c/BW0bbP/71OAF). In our collection, we also identified this S-subunit (WP_002287733) as present and enriched in clade A1 isolates (Fisher’s exact test, P < 0.05). As previously reported [(6)](https://paperpile.com/c/BW0bbP/71OAF), the subunits M and R were present in both clade A1 and non-clade A1 isolates, which suggests that the specificity of the system resides in the subunit S which binds to different DNA sequences. Based on this, we further explored and identified 8 novel S-subunits variants which were present in our set of 62 isolates with complete genomes. The multiple sequence alignment of the unique S-subunit variants (8 novel variants and reference S-subunit variant (accession number WP_002287733)) found in our set of completed genomes is available at: <https://gitlab.com/sirarredondo/efaecium_population/tree/master/Files/rmsystems>

Several S-subunits were enriched in clade A1 isolates (E1774_00555, E7313_02981 , E4413_00571, E4438_00276) and mainly present in hospitalized patients and dog isolates. In contrast, S-subunit variants E0139_00520 and E4227_02943 were enriched in non-clade A1 isolates. These observations may indicate that different sets of RM systems in *E. faecium* population have contributed to the differentiation of the plasmidome content depending on the isolation source.

**Suppl. Results: Plasmidome populations are strongly associated with isolation source**

In the following section, we unravel and provide a detailed characterization of the genes present in each plasmidome population. We first assess whether there is a particular *E. faecium* isolation source significantly overrepresented in the population and whether it exists a high SC diversity of the isolates which could indicate horizontal transmission of plasmid sequences (Figure 3B, Suppl. Figure 4B and Figure S5). To identify which genes are driving these populations, we defined the plasmidome-population core genes (present > 95% isolates) and characterized their COG and manually curated and searched some of their functions in literature. We finally compared the sequences to our set of complete plasmid sequences to match the type of plasmid replicons bearing these plasmidome-population core genes.

Some of the plasmidome-population core genes defined below were present in several populations. In this analysis, we tried to define which genes are commonly present in the isolates from a particular population. The presence of a plasmidome-population core gene for a particular population does not imply the absence of that gene in another population, as not a single, but the pool of plasmid genes are the ones defining the population. Plasmidome-population core genes shared between populations were mainly involved in plasmid replication, recombination, repair, mobilization or stabilization. These mechanisms are used by plasmid sequences and belong to the backbone of plasmids and thus can be found in populations with overrepresentation of different hosts (e.g. poultry and dog population).

The pangenome of each population and the COG annotation generated by eggNOG are available at: <https://gitlab.com/sirarredondo/efaecium_population/tree/master/Files/Plasmid_populations>

In the sections below we refer to the plasmidome-population genes using a locus tag present each pangenome fasta file provided at : <https://gitlab.com/sirarredondo/efaecium_population/tree/master/Files/Plasmid_populations> . The complete annotation of each plasmidome core gene with each associated locus tag is also available in Supplementary Dataset S1.

Population 1

Plasmidome population 1 was enriched for pig and non-hospitalized person isolates (Bonferroni-corrected *P* < 0.05) suggesting transmission of plasmid sequences between these two sources. We confirmed this by inspecting our set of completed plasmid sequences and chromosomes. Isolate E0139 (from a non-hospitalized person) and isolate E0595 (derived from a pig) shared a near-identical (99.4% identity, 96% coverage) RepA_N conjugative plasmid of 140 kbp (accession numbers LR132068.1 and LR135180.1) which suggested an exchange of this plasmid between these different source types. However, the corresponding chromosome of isolates E0595 and E0139 exhibited different SC’s (30 and 29), indicating that the presence of this identical plasmid in these two isolates is the result of horizontal transfer of plasmids, rather than vertical transfer. In general, plasmidome population 1 had a large diversity of SC’s (Simpson index = 0.53, CI = 0.524-0.630) suggesting horizontal spread of at least a part of the plasmid sequences defining this population.

We identified a total of 111 genes present as part of the core-plasmidome this population. From these 111 genes, only 68 had an associated COG. The most predominant COG was the category S (unannotated function) with 18 genes. We manually inspected these genes and predicted their potential function which included a i) toxin-antitoxin component (TA) system corresponding to the toxin belonging to RelE and AbrB transcriptional regulator (IIAENCCH_00121, IIAENCCH_00120, and a toxin component of the Fic family (IIAENCCH_00140), ii) Abi system formed by the AbiEi (IIAENCCH_00016) and AbiEii (IIAENCCH_00017), iii) a starvation protective gene against oxidative damage (IIAENCCH_00129) and iv) an ABC transporter permease FetB, exporting iron (IIAENCCH_00146).

These groups include genes involved in mechanisms of plasmid stabilization such as TAs that may explain the persistence of large plasmids in the population in the absence of a selective pressure. Furthermore, TA systems have been postulated as attractive targets to stop the dissemination of vancomycin resistance in *E. faecium* [(7, 8)](https://paperpile.com/c/BW0bbP/CthLD+Al9K2). The AbiEi/AbiEii system described corresponds to an innate immune mechanism that provides viral protection against phage dissemination and its mechanism of action interferes with phage RNA synthesis and also enable stabilization of mobile genetic elements [(9)](https://paperpile.com/c/BW0bbP/61xqz). Interestingly, this system has been extensively described in lactococcal plasmids [(10)](https://paperpile.com/c/BW0bbP/Dsvjn).

The following most predominant COG groups corresponded to COG L (14 genes) and COG M (10 genes). COG L genes belonged mainly to genes involved in plasmid replication, recombination and repair such as ISEfa7 transposase (IIAENCCH_00090), IS1476 transposases (IIAENCCH_00105, IIAENCCH_00106) ISEnfa3 transposase (IIAENCCH_00109) or DNA topoisomerase III (IIAENCCH_00019) among other examples (Suppl. Dataset S1).

In the COG M group, we detected a copper resistance gene operon (*tcrYAZB* operon) (IIAENCCH_00096, IIAENCCH_00107, IIAENCCH_00137, IIAENCCH_00139, IIAENCCH_00095, IIAENCCH_00136) that mediates resistance against this heavy-metal and was previously described in *E. faecium* as plasmid-borne [*(11)*](https://paperpile.com/c/BW0bbP/JXJ08). Copper was commonly used as a growth-promoting agent to increase pig production [(11)](https://paperpile.com/c/BW0bbP/JXJ08). However, high-levels of copper result is toxic for the cells. The *tcrYAZB* operon provides a plasmid-survival mechanism to tolerate high concentrations of this heavy-metal.

We found that the glycopeptide resistance gene *vanA* (IIAENCCH_00115) was also part of the set of plasmidome-population core genes of this population. Furthermore, we also identified an ATPase from a type IV secretion system (IIAENCCH_00032) and the TraG family conjugation protein (IIAENCCH_00037) as a plasmidome-population core gene which suggests the presence of an active and widespread system to enhance plasmid mobilization.

The plasmidome-population core genes described above were present in the complete plasmid sequences previously mentioned corresponding to a RepA_N conjugative plasmid (accession numbers LR132068.1 and LR135180.1). The introduction of this plasmid in the population together with the pool of genes described could be explained due to selective pressures such as high concentrations of copper or the usage of glycopeptides during pig breeding. The presence of TA systems or Abi systems such as AbiEi/AbiEii may play a role in stabilizing the plasmid structure after removing the initial selection pressure.

Population 2

Plasmidome population 2 was significantly overrepresented by poultry isolates (Bonferroni *P* < 0.05) and exhibited a high homogeneity of SC’s (Simpson index = 0.21, CI = 0.210-0.359) suggesting that plasmid sequences within this population were mainly vertically inherited.

We identified a total of 93 genes as belonging to the core-plasmidome of population 2 and 58 genes had an associated COG category. The most predominant COG group corresponded to COG L with a total of 14 genes which included plasmid replication, recombination and repair genes such as DNA topoisomerases III (GDKHCPLE_00029, GDKHCPLE_00091), IS66 Orf2-like proteins (GDKHCPLE_00142), HNH endonuclease (GDKHCPLE_00092) among other examples (Suppl. Dataset S1).

The second most predominant group was COG G which included mainly genes associated to carbohydrate utilisation such as PTS systems involved in: i) trehalose/maltose utilisation (GDKHCPLE_00038, GDKHCPLE_00109), ii) PTS system involved in N-acetylglucosamine (GDKHCPLE_00037), iii) glycosyl hydrolase (GDKHCPLE_00105) or iv) fructokinases (GDKHCPLE_00104, GDKHCPLE_00110) among other examples. This may confer novel pathways for carbohydrate usage in this poultry-associated population.

We detected a BSH choloylglycine hydrolase member (GDKHCPLE_00140, COG M) as being part of the core of this plasmidome populations. The role of BSH activity in the intestine of poultry is unclear but it has been hypothesized that can confer tolerance to the bile [(12)](https://paperpile.com/c/BW0bbP/4ar3R). Again, we find glycopeptide resistance mediated by *vanA* (GDKHCPLE_00050) within the set of core-genes in this population. We could also identify an additional resistance gene corresponding to a streptomycin adenyltransferase (*aadE*, GDKHCPLE_00054).

Also TA systems mediated by RelE toxin (NGNCBCCO_00115) and an ATPase from a type IV secretion system (GDKHCPLE_00017) which may enhance the mobilization of plasmid sequences, were part of the core genes in this plasmid population.

We identified as plasmidome-population core a tetronasin resistance gene (GDKHCPLE_00084). The presence of this tetronasin resistance gene on mobile element among *E. faecium* poultry isolates has been previously described and may be related to the widely use of ionophores, e.g. tetronasin for coccidiosis prophylaxis in poultry [(13)](https://paperpile.com/c/BW0bbP/AWQUH).

In our pool of complete plasmid sequences, we observed the presence of two near-identical plasmids present in the poultry isolates E4227 and E4239 which were sequenced to completion: i) a RepA_N conjugative plasmid (175 kbp) corresponding to accession numbers LR135171 and LR135783 (100% identity and 100% coverage) and ii) multireplicon Inc18 and Rep3 plasmid (46 kbp) corresponding to accession numbers LR135172 and LR135784 (100% identity and 99% coverage) .

Population 3

This population was significantly overrepresented with hospitalized patient isolates. SC diversity measured by the Simpson index (0.04, CI = 0.037-0.110) indicated that the isolates belonging to this population shared plasmid sequences which were mainly transmitted due to vertical inheritance.

In this population, we identified a total of 73 plasmidome-population core genes from which 37 had an associated COG. The most predominant COG corresponded to the category S (unknown function) with 13 genes. Within this group, i) TA system formed by the Txe/YoeB module (GCEJCPEH_00117, GCEJCPEH_00118) and ii) a duplicated Abi system formed by AbiEi (GCEJCPEH_00086, OLDHMAJC_00207) and AbiEii (GCEJCPEH_00085, OLDHMAJC_00206) were identified.

The second most predominant COG corresponded to COG L with a total of 7 genes and mainly corresponding to genes involved in plasmid replication, recombination and repair such as DNA topoisomerase III (GCEJCPEH_00088), helix-destabilizing (GCEJCPEH_00084) or resolvase proteins (OLDHMAJC_00341) among other examples (Suppl. Dataset S1). We identified three genes categorized as COG V (defense mechanism) corresponding to an ABC transport system formed by an ATP-binding protein (OLDHMAJC_00277) and two permeases (OLDHMAJC_00276, OLDHMAJC_00278). These three genes are similar to the previously described *vex* locus in *Streptococcus pneumoniae* [*(14)*](https://paperpile.com/c/BW0bbP/68vCp). As plasmidome-population core genes, we identified an ATPase involved in a type IV secretion system (GCEJCPEH_00068) and the TraG family conjugation protein (GCEJCPEH_00063) which may contribute to the mobilization and spread of plasmid sequences in the population, and the *erm* gene (GCEJCPEH_00113) conferring resistance to macrolide, lincosamide and streptogramin B.

We inspected our set of complete plasmid sequences derived from long-read isolates (E7196, E7654 and E7663) belonging to the population 3. We found three plasmid structures bearing the described plasmidome-population core genes: i) a RepA_N plasmid with a length higher than 160 kbp (LR135271, LR135325, LR135318), ii) a different RepA_N plasmid with a length around 62 kbp (LR135272, LR135326, LR135319) and iii) a multireplicon plasmid Inc18 & Rep_1 only present in the isolates E7654 (LR135327) and E7663 (LR135320) with a length around 38 kbp.

Population 4

Plasmidome population 4 was enriched among dog isolates (Bonferroni corrected P < 0.05) with a high SC diversity (Simpson index = 0.73, CI = 0.728-0.781) suggesting horizontal spread of plasmid sequences between isolates from this population.

We found a total of 27 genes as part of the core-plasmidome of population 4 from which 17 genes had an associated COG (Supplementary Dataset S1). The most predominant COG corresponded to the unknown function S with 8 genes; i) TA systems formed by the toxin RelE (HBJAKGIG_00118) and a consecutive antitoxin component from the AbrB family (HBJAKGIG_00119), plus another the toxin component of the Fic family (HBJAKGIG_00176) ii) a starvation protective gene against oxidative damage, DPS protein (HBJAKGIG_00170), iii) Abi system formed by AbiEi (HBJAKGIG_00149) and AbiEii (HBJAKGIG_00148). Two plasmidome-population core genes belonging to the category COG G, represented a predicted PTS systems, a sucrose-6-phosphate hydrolase (HBJAKGIG_00172) and a subunit of a beta-glucoside transporter (HBJAKGIG_00173).

We found an ATPase part of a type IV secretion system (HBJAKGIG_00052) which could enhance the mobilization of plasmid sequences in the population. The lower number of genes (n = 27) present in the core-plasmidome respect to other populations may indicate that isolates belonging to this population present a higher heterogeneity of plasmid content.

Two hospitalized patient isolates (E8040 and E8172) clustered in this dog plasmidome population. E8172 was long-read sequenced and contained a conjugative RepA_N plasmid (156 kbp, accession number LR135373.1) with high levels of similarity but some structural rearrangements (99.9% identity, 77% coverage) when compared to another conjugative RepA_N plasmid (148 kbp, accession number LR135259.1) present in the completely sequenced dog isolate E4457 from the same plasmidome population. Similar RepA_N plasmids are found in other long-read isolates present in the population such as E4402 (accession number LR135175, 149 kbp), E4413 (accession number LR135186, 172 kbp), E8481 (accession number LR536671, 149 kbp) and E4438 (accession number LR135192, 145 kbp).

Population 5

This population was significantly overrepresented by hospitalized patient isolates. SC diversity measured by the Simpson index (0.72, CI = 0.720-0.777) suggested horizontal transmission of plasmid sequences in isolates belonging to this population.

We found a total of 152 plasmidome-population core genes from which 106 had an associated COG. There two most predominant COG groups with 25 genes respectively, COG S (unknown function) and COG L (replication, recombination, repair). COG S included the following TA systems: i) a RelE (AAEHJEFK_00145, AAEHJEFK_00224) and AbrB (AAEHJEFK_00144, AAEHJEFK_00223) system, ii) a Txe/YoeB module (AAEHJEFK_00221,AAEHJEFK_00222), iii) a HicA/HicB module (AAEHJEFK_00072, AAEHJEFK_00073), iv) toxin component of the Fic family (AAEHJEFK_00035) and v) MazE/MazF system (AAEHJEFK_00121,AAEHJEFK_00122). MazE had not COG associated but was defined as plasmidome-population core gene in the population.

From COG L, included a variety of genes such as IS1216 transposase (AAEHJEFK_00150), IS256 transposase (AAEHJEFK_00252), ISEf1 transposase (AAEHJEFK_00199), DNA topoisomerase III (AAEHJEFK_00243), resolvases (AAEHJEFK_00079) among other examples (Suppl. Dataset S1). Three genes constituted the *panBCD* (AAEHJEFK_00201, AAEHJEFK_00202, AAEHJEFK_00203) locus, previously described in *Streptococcus gallolyticus* and that encodes for the complete biosynthetic pathways of panthotenate. This locus may provide a selective advantage of *E. faecium* isolates containing this locus to outcompete and grow in an environment with a variety of carbohydrates and poor amino acid source [(15)](https://paperpile.com/c/BW0bbP/Hfypr).

Other plasmidome-population core genes putatively encode a peptidoglycan binding domain protein (COG M, AAEHJEFK_00157) with two domains implicated in: i) peptidoglycan binding and ii) glycoside hydrolase superfamily (GH25_BacA-like). We searched the protein sequence against *BacA* homologues that were previously described as a plasmid-encoding bacteriocin in *E. faecalis* [*(16)*](https://paperpile.com/c/BW0bbP/fsSUF). *BacA* homologues are splitted into five different clades [(16)](https://paperpile.com/c/BW0bbP/fsSUF). In our study, we observed a perfect match (blastp, e-value = 0.0, identity = 99%) between AAEHJEFK_00157 and EOK45589 which belongs to clade IV variant. We could not identify other Bac41-like genes in the adjacent areas of *BacA*, which is in accordance with the findings of *BacA* clade IV described by Kurushima et al 2016. Furthermore the authors argue about the functionality of this *BacA* homologue gene since they showed that the presence of *BacL1* (another Bac41-like gene) is required for bacteriolysin activity. Kurushima et al. 2016 described that *BacA* gene can act as a more evolved toxin-antitoxin system in which not only daughter cells but also cells from the same generation not bearing the plasmid gene are excluded. Furthermore, the authors showed that plasmid dissemination was more prominent under conditions of *E. faecium* populations fluctuations since the gene activity exclusively affects dividing cells.

We again find the locus of three genes corresponding to an ABC transport system formed by an ATP-binding protein (AAEHJEFK_00219) and two efflux ABC transport systems (AAEHJEFK_00218, AAEHJEFK_00220) acting as permeases which was previously described in *S. pneumoniae* as *vex* locus. We also detected the TraG gene (AAEHJEFK_00015, COG U) encoding for a conjugation protein and an ATPase from a type IV secretion system (AAEHJEFK_00020), which may indicate that the horizontal transmission of plasmid sequences within this population is mediated by this conjugation system.

Several antimicrobial resistance genes were part of the core in plasmidome population 5 including: i) aminoglycoside resistance (*aacA-aphD* gene) (AAEHJEFK_00194), ii) macrolide, lincosamide and streptogramin B resistance (*erm* gene) (AAEHJEFK_00107), iii) glycopeptide resistance (*vanA* gene) (AAEHJEFK_00234) and iv) teicoplanin resistance (AAEHJEFK_00142).

We detected a RepA_N plasmid with a length around 165 kbp shared between the isolates E6043 (accession number LR134106), E7040 (accession number LR135220) and E7067 (accession number LR135236). Secondly, we observed a RepA_N like plasmid present also in these E6043 (accession number LR134108), E7040 (accession number LR135222), E7067 (accession number LR135238) and E7207 with a length around 55 kbp and containing the TraG gene described before. And lastly, we identified a multireplicon Inc18 & Rep_3-like plasmid shared between the isolate E6043 (accession number LR134110), E7067 (accession number LR135239) and E7040 (accession number LR135223) with structural rearrangements and length ranging from 38 kbp to 52 kbp. The aminoglycoside resistance gene (AAEHJEFK_00194) was located in the RepA_N plasmid of 165 kbp whereas the other resistance genes (*erm*, *vanA* and teicoplanin resistance gene) were carried by the multireplicon Inc18 & Rep_3-like plasmid. The existence of different plasmid replicons present in the population underpins the importance of analysing the entire pool of plasmid genes rather than focusing on an individual plasmid replicons.

Population 6

Population 6 was significantly overrepresented by hospitalized patient isolates. Based on the heterogeneity of SC’s (Simpson index = 0.30, CI = 0.299-0.422) , we concluded that the plasmid sequences present in the population were mainly vertically inherited since most of the isolates of the population belonged to a narrow range of SC’s.

In total, we identified 128 plasmidome-population core genes from which 86 had an associated COG. We observed again that the most predominant COG group corresponded to the category S (unknown function) (Suppl. Dataset S1). Core genes in this population represent i) toxin RelE (LDCOMLJG_00062) and antitoxin system (LDCOMLJG_00063) from AbrB family, TA system MazE/MazF (LDCOMLJG_00194, LDCOMLJG_00195) and a toxin component from Fic family (LDCOMLJG_00190), ii) Abi system formed by AbiEi (LDCOMLJG_00151) and AbiEii (LDCOMLJG_00152).

The following most predominant group was COG L which included ISL3 transposase (LDCOMLJG_00028), IS256 transposase (LDCOMLJG_00214), IS200 transposase (LDCOMLJG_00269) or a DNA topoisomerase III (LDCOMLJG_00149) among other examples.

Eight genes grouped within the category COG G were encoding among other functions for a complete PTS system involved in mannose/fructose/sorbose utilisation: i) IIA component (LDCOMLJG_00079), ii) IIB (LDCOMLJG_00080), iii) IIC (LDCOMLJG_00081), and iv) IID (LDCOMLJG_00082). We also found the TraG conjugation protein (LDCOMLJG_00222, COG U) present as plasmidome-population core gene suggesting the presence of a conjugative plasmid widely spread in this population.

Also the *erm* gene (macrolide, lincosamide and streptogramin B resistance) (LDCOMLJG_00008) belonged to the plasmidome-population core. The locus of three genes encoding for an ABC transporter system including two efflux ABC transporters acting as permeases (LDCOMLJG_00181, LDCOMLJG_00183) and an ATP-binding protein (LDCOMLJG_00182) also belonged to the core in this population.

We confirmed the presence of the plasmidome-population core genes in our set of complete plasmid sequences derived from long-read sequenced isolates belonging to population 6 (accession numbers for E8202; LR135345, E7429; LR135298, E6055; LR135198, E7356; LR135340 and E8195; LR135365). We observed a highly similar RepA_N plasmid but showing structural rearrangements depending on the isolates and carrying most of the previously described plasmidome-population core genes.

Population 7

This population was also hospital-associated and its SC diversity (Simpson index = 0.56, CI = 0.561-0.649) suggested horizontal transmission of the plasmid sequences in the population. We identified a total of 138 plasmidome-population core genes from which 86 had an associated COG. In this case, the most predominant COG group corresponded to the category G (carbohydrate transport and metabolism).

This group included a complete set of PTS systems involved in mannose/fructose/sorbose-specific utilisation consisting of: i) IIA components (FIBMOKAC_00130, FIBMOKAC_00193), ii) IIB components (FIBMOKAC_00129, FIBMOKAC_00194), iii) IC components (FIBMOKAC_00128, FIBMOKAC_00195), iv) IID components (FIBMOKAC_00127, FIBMOKAC_00196), and v) glycosyl hydrolases (FIBMOKAC_00134), sugar kinases (FIBMOKAC_00257) (Suppl. Dataset S1). This highlights that most of the defined plasmidome-population core genes in this population are responsible for the utilisation of complex carbohydrates.

In this population, we also identified several TA systems catalogued as plasmidome-population core genes: i) MazE/MazF system (FIBMOKAC_00110, FIBMOKAC_00109), ii) toxin component of the Fic family (FIBMOKAC_00062) and iii) toxin RelE (FIBMOKAC_00122).

We observed a plasmidome-population core gene encoding a peptidoglycan binding domain protein (FIBMOKAC_00168) and with similarity to *BacA* belonged to the plasmidome-population core in population 7. Furthermore, we identified the set of three genes forming the vex locus previously mentioned, formed by two efflux ABC transporters acting as permeases (EIAGHGLI_00196, EIAGHGLI_00200) and an ATP-binding protein (EIAGHGLI_00196). We also observed the presence of an ATPase from a type IV secretion system (FIBMOKAC_00012) that could be involved in the mobilization of plasmid sequences.

We could confirm the widespread of these plasmidome-population core genes by inspecting our set of completed genome isolates from long-read sequenced isolates (n = 4) corresponding to hospitalized patients (E7313, E8014 and E8423) and belonging to plasmidome population 7. These isolates shared an identical RepA_N plasmid (> 200 kbp) with some structural rearrangements between their sequences and bearing the same set of PTS system present in two different parts of the plasmid replicon. To highlight this observation, we focused on the isolates E8014 and E8423 belonging to the SC groups 13 and 18 respectively. Both isolates carried a similar large RepA_N plasmid sequence (accession numbers LR135352 and LR135476, 99.7% identity and 86% coverage, length > 200kbp) despite being non-clonally related.

Population 8

This population was also overrepresented among hospitalized patients but its associated Simpson index (0.15, CI = 0.151-0.216) indicated that the plasmid sequences present in this population were mainly clonally inherited. In total, we found 138 plasmidome-population core genes from which 88 had an associated COG. The most predominant COG group was the category L (replication, recombination, repair) with a total of 21 genes. Within this group, we found DNA topoisomerases III (EIAGHGLI_00076, EIAGHGLI_00227), ISEnfa3 transposases (HGPANJKB_00188, HGPANJKB_00238) or an IS256 transposase (EIAGHGLI_00195) among other examples (Suppl. Dataset S1). The second most predominant group (20 genes) represented category S (unknown function). Here, we could group genes in two classes: i) TA systems, including RelE/AbrB (EIAGHGLI_00212,EIAGHGLI_00211), MazEF (EIAGHGLI_00255, EIAGHGLI_00256) and Xre antitoxin component (EIAGHGLI_00223) and ii) Abi system formed by AbiEi and AbiEii (HGPANJKB_00033, HGPANJKB_00034) .

We also found 12 plasmidome-population core genes belonging to COG category G which were mainly predicted to encode PTS systems: i) N-acetylglucosamine-specific IIABC component (EIAGHGLI_00204), ii) trehalose/maltose-specific IIBCA component (EIAGHGLI_00205) and iii) lactose/cellobiose-specific IIC component (EIAGHGLI_00221), among other examples of carbohydrate degradation and transport (Suppl. Dataset S1).

We identified the same two efflux ABC transporters acting as permeases (EIAGHGLI_00196, EIAGHGLI_00200) and an ATP-binding protein (EIAGHGLI_00197) as well as the peptidoglycan binding domain protein (EIAGHGLI_00233) with highly similarity to BacA as plasmidome core genes.

Also an ATPase gene from a type IV secretion system (EIAGHGLI_00030, COG U) and the antimicrobial resistance gene, *erm* gene (EIAGHGLI_00163) conferring macrolide, lincosamide and streptogramin B resistance were assigned as plasmidome core genes.

We confirmed the presence of these plasmidome-population core genes in the complete plasmid sequences derived from long-read isolates belonging to population 8. The genes were carried into two different complete plasmid structures: i) a RepA_N plasmid with a length around 240 kbp present in the long-read isolates E8290 (accession number LR135395), E8414 (accession number LR135489), E7933 (accession number LR135385), E8328 (accession number [LR135415](https://www.ebi.ac.uk/ena/data/view/LR135415)), E8284 (accession number [LR135409](https://www.ebi.ac.uk/ena/data/view/LR135409)) and E8377 (accession number [LR135402](https://www.ebi.ac.uk/ena/data/view/LR135402)) and ii) an Inc18 plasmid with a length around 63 kbp present in the long-read isolates E8290 (accession number [LR135396](https://www.ebi.ac.uk/ena/data/view/LR135396)), E8414 (accession number [LR135491](https://www.ebi.ac.uk/ena/data/view/LR135491)), E7933 (accession number [LR135387](https://www.ebi.ac.uk/ena/data/view/LR135387)), E8284 (accession number [LR135410](https://www.ebi.ac.uk/ena/data/view/LR135410)) and E8377 (accession number [LR135403](https://www.ebi.ac.uk/ena/data/view/LR135403)). Most of the plasmidome-population core genes resided in the RepA_N plasmid (240 kbp) whereas the *erm* gene and Xre toxin/antitoxin system was present in the Inc18 plasmid.

Population 9

This population was significantly associated to hospitalized patients and its SC diversity (Simpson index = 0.04, CI = 0.037-0.110) indicated that the plasmid sequences shared in the isolates belonging to the population were mainly vertically inherited. This population had the largest number of plasmidome-population core genes with a total of 205 from which 134 had an associated COG. There were two predominant COG groups L (replication, recombination and repair) and S (unknown function) which 34 genes respectively. Within COG S group, we identified a similar set of genes and included the following categories: i) TA systems, RelE/AbrB system (OCOMIPFD_00142, OCOMIPFD_00141), MazEF system (OCOMIPFD_00214, OCOMIPFD_00215), HicAB system (OCOMIPFD_00079, OCOMIPFD_00080), toxin component of the Fic family (OCOMIPFD_00039), Txe/YoeB module (OCOMIPFD_00114, OCOMIPFD_00113) and ii) Abi system formed by AbiEi (OCOMIPFD_00180) and AbiEii (OCOMIPFD_00179).

In COG group L, we identified several transposases such as ISEfa8 (OCOMIPFD_00144), ISEf1 (OCOMIPFD_00151), ISEfa7 (OCOMIPFD_00197) transposases, DNA topoisomerases III (OCOMIPFD_00168), among other examples (Suppl. Dataset S1).

We again observed the presence of an ABC transporter system formed by two permeases (OCOMIPFD_00186, OCOMIPFD_00188) and an ATP-binding protein (OCOMIPFD_00187) as plasmidome core gene. In this population, we also observed a large set of antimicrobial resistance core genes including: i) aminoglycoside resistance genes, *aacA-aphD* (OCOMIPFD_00249), *aadE* (OCOMIPFD_00201) and *aphA* (OCOMIPFD_00271), ii) chloramphenicol resistance, *cat* gene (OCOMIPFD_00281), iii) macrolide, lincosamide and streptogramin B resistance, *erm* gene (OCOMIPFD_00086) and iv) glycopeptide resistance, *vanA* gene (OCOMIPFD_00136).

**Suppl. Results: plasmidome-population core genes present in hospitalized patient isolates**

In the previous section, we described the plasmidome-population core genes present in each population. Next, we analyze which plasmidome core genes were found among all the hospitalized patient isolates. This revealed only 10 plasmidome-population core genes present, which had an associated COG annotation. These genes corresponded to: i) antitoxin component from the AbrB family (OMLCIILE_00043) , ii) replication-associated protein (OMLCIILE_00041), iii) replication initiator protein (OMLCIILE_00049), iv) single-strand binding protein, ssb (OMLCIILE_00299), v) tyrosine recombinase, XerS (OMLCIILE_00437) and vi) putative transposon Tn*552* DNA-invertase bin3 (OMLCIILE_00343). These genes are mainly involved in plasmid replication, recombination, repair or stabilization and thus are present within different plasmid structures. The low number of plasmidome core genes among hospitalized patient isolates may be explained by the heterogeneity (differences in isolation time, year and countries) of isolates falling under this category and emphasizing that different plasmid configurations have evolved within this source group.

If we lower the threshold to define a gene as plasmidome-population core (from 95% to > 90% isolates) we identified 39 plasmidome-population core genes from which 22 had an associated COG annotation. This set of 39 plasmidome-population core genes (present in > 90% of hospitalized patient isolates), included several genes previously highlighted for some of the populations, like antimicrobial resistance genes such as aminoglycoside resistance (*aacA-aphD*, OMLCIILE_00488) and ii) erythromycin resistance (*erm*, OMLCIILE_00367) and the RelE toxin (OMLCIILE_00042). RelE is frequently coupled with the antitoxin component from the AbrB family described above, which makes this TA system an attractive target to combat antimicrobial resistance in hospitalized patients by plasmid clearance. Furthermore, we observed the locus of three genes formed by two permeases (HKLEHDKC_00083, OMLCIILE_00480) and an ATP-binding protein (OCOMIPFD_00187). This locus of three genes was not present in populations not related to hospitalized patients (population 1, 2 and 4) which suggests that these three genes may have contributed to the adaptation of *E. faecium* to the hospital environment. We also identified two genes described several times in the previous populations related to mobilization of plasmid sequences formed by a type IV secretion system (OMLCIILE_00070) and TraG involved in conjugation machinery (OMLCIILE_00070).

Supplementary Text (Methods)

# **Suppl. Methods: Illumina sequencing**

Bacterial isolates were grown overnight (O/N) at 37°C on blood agar plates. Single colonies were picked up and grown O/N at 37°C with Brain Heart Infusion (BHI). Bacterial cell pellets were pretreated and incubated 1-4 hours with 180 µL of enzymatic lysis buffer. Subsequently, 0.75 mg proteinase K were added and incubated at 56°C until lysis completion. 20 µL of RNAse A (10mg/mL) were added and incubated for 5’ at room-temperature (RT). Total DNA purification was performed using and following the protocol from NucleoSpin 96 Tissue Core Kit (Machery-Nagel), vacuum processing. DNA concentration was measured using Quant-it Picogreen (Thermo Fisher Scientific). Library preparation was carried out following Nextera DNA Library Prep Reference Guide. Finally, Nextera libraries were sequenced using Illumina NextSeq at USEQ, Utrecht, The Netherlands (http://www.useq.nl).

### **Suppl. Methods: WGS short-read assemblies**

Illumina reads were trimmed using nesoni clip, part of the nesoni toolkit (version 0.132), with the following settings: ‘--adaptor-clip yes --match 10 --max-errors 1 --clip-ambiguous yes --quality 10 --length 90 --trim-start 0 --trim-end 0 --gzip no --out-separate yes pairs:’. Trimmed reads were then assembled into scaffolds using SPAdes (version 3.5.0) with default settings. Scaffolds with an average coverage lower than 10 and/or a length smaller than 500bp were removed from the assemblies.

### **Suppl. Methods: Selection of isolates to sequence by ONT**

A fraction (n=62) of the total number of isolates was selected for long-read sequencing using Nanopore technology. We initially predicted the plasmid content of the isolates *in silico* using *PlasmidSPAdes* (version 3.8.2) which performs *de novo* assembly filtering out contigs with a coverage similar to the host chromosome coverage [(17)](https://paperpile.com/c/BW0bbP/fNwr8). *Prokka* (version 1.12) was used to annotate the putative remaining plasmid contigs specifying the custom *Enterococcus* database provided [(18)](https://paperpile.com/c/BW0bbP/R5NFc). Orthologous clustered genes were estimated using *Roary* (version 3.8), splitting paralogues and defining a threshold of 95% amino-acid level similarity to cluster protein sequences [(19)](https://paperpile.com/c/BW0bbP/Ahu3k). This multi-dimensionality matrix was then reduced and visualized to two dimensions using the t-Distributed Stochastic Neighbor Embedding (*t-SNE*) (theta = 0.5, iterations = 1000, dims = 2) using the implementation provided in the *R* package *Rtsne* (version 0.13) [(20, 21)](https://paperpile.com/c/BW0bbP/3Gjne+ow7DZ). To avoid manual selection of the isolates, k-means function (iter.max = 1000) provided in the *R* package stats (version 3.4.4) was used to and allocated 50 centroids into the dimensionality reduced distribution given by *tSNE. Euclidean* distance of each isolate was calculated to extract the 50 isolates closest to each centroid.

To cover all plasmid replication genes not present in the first selection, 12 additional isolates were selected for Nanopore sequencing. This second selection was based on a reciprocal blast (blastx and tblastn, -evalue 1e-10) of the predicted plasmid orthologous genes against 76 previously described plasmid replication amino-acid sequences from the genus *Enterococcus* [*(22)*](https://paperpile.com/c/BW0bbP/GiXNg). Isolates bearing plasmid replication genes not present in the first selection were sorted and selected based on the highest number of orthologous genes.

### **Suppl. Methods: ONT sequencing**

*E. faecium* selected isolates (n = 62) were grown O/N at 37°C on blood agar plates, then single colonies were picked up and grown with BHI at 37°C. Genomic DNA was extracted using the Wizard Genomic DNA purification kit (Promega) following manufacturer’s instructions. Isolated DNA was sheared (4000 rpm, 2x120 seconds) using G-tubes (Covaris). Library preparation was performed using Ligation Sequencing Kit 1D (SQK-LSK108) with the Native Barcoding Kit 1D (EXP-NBD103). Genomic libraries were loaded onto R9.4 (FLO-MIN106) flowcells using the MinION device (Mk2). Libraries were basecalled using Metrichor workflows (Run 1 ,2, 3), Albacore 1.01 (Run 4, 5) and Albacore 1.1.0 (Run 6). ONT Sequencing and basecalling were conducted at USEQ, Utrecht, The Netherlands (http://www.useq.nl)

### **Suppl. Methods: Assembly of ONT sequenced isolates**

Fastq files were obtained from base-called data using Poretools (version 0.6.0) except for Run6 in which fastq files were retrieved using Albacore (version 1.1.0). Distribution of read length and total number of reads were calculated using Bioawk (version 20110810, <https://github.com/lh3/bioawk>). We used Porechop (version 0.2.1, <https://github.com/rrwick/Porechop>) to trim reads and filter out chimeras from different bins specifying the flag “--discard_middle”. Illumina reads were trimmed using seqtk (version 1.2-r94, <https://github.com/lh3/seqtk>) with the command “--trimfq” prior to assembly.

Hybrid assembly was performed using Unicycler (version 0.4.1), specifying “bold” mode [(23)](https://paperpile.com/c/BW0bbP/orEgO). Briefly, Unicycler uses SPAdes (version 3.6.2) to create different assembly graphs based on different k-mer size only considering Illumina reads [(24)](https://paperpile.com/c/BW0bbP/xs5Pb). The best assembly graph was selected by Unicycler based on number of dead-ends and contiguity. Next, all ONT reads were used to scaffold and solve the assembly graph. Additionally, we specified the same file as described above (section ‘*Selection of isolates to sequence by ONT’*) containing 76 known plasmid replication sequences to rotate and change the 0-coordinate of circular replicons resulting from hybrid assembly [(22)](https://paperpile.com/c/BW0bbP/GiXNg). Finally, Unicycler conducted several rounds of Pilon (version 1.22) to polish genome sequences using Illumina reads [(25)](https://paperpile.com/c/BW0bbP/0gumb).

### **Suppl. Methods: Characterization of fully assembled plasmids**

Contigs derived from hybrid assembly were labeled either as chromosome or plasmid based on sequence length and circularization signatures. Contigs were categorized as plasmid if they presented circularization signatures and a sequence length smaller than 350 kbp. Putative plasmids smaller than 350 kbp and lacking circularization signatures were not considered for further analysis. Rapid annotation by Prokka (version 1.12) [(18)](https://paperpile.com/c/BW0bbP/R5NFc) allowed us to discard four putative circular phage sequences.

We used Abricate (version 0.8.2) to query (> 80% identity & > 60% coverage) our set of completed plasmid sequences (n = 305) versus a curated database of known replication initiator and relaxases proteins from *Enterococcus* [*(5, 26)*](https://paperpile.com/c/BW0bbP/QkWvX+o19PW). Replicon sequences with a lower identity (< 80% ID) were classified as Rip-like.

Completed plasmid sequences were clustered using Mash (k = 21, s = 1000 ; version 1.1) [(27)](https://paperpile.com/c/BW0bbP/HtwJD) and the resulting distance matrix was clustered using the hclust function (method = ‘ward.D2’) provided in R package stats (version 3.4.4). Dendrogram visualization and metadata associated to plasmid isolates was displayed using ggtree (version 1.13.3) [(28)](https://paperpile.com/c/BW0bbP/AJqjG).

# Supplementary Text References

1. [Teuber M, Schwarz F, Perreten V. 2003. Molecular structure and evolution of the conjugative multiresistance plasmid pRE25 of Enterococcus faecalis isolated from a raw-fermented sausage. Int J Food Microbiol 88:325–329.](http://paperpile.com/b/BW0bbP/e0vxc)

2. [Garcia-Migura L, Hasman H, Jensen LB. 2009. Presence of pRI1: a small cryptic mobilizable plasmid isolated from Enterococcus faecium of human and animal origin. Curr Microbiol 58:95–100.](http://paperpile.com/b/BW0bbP/p3TIJ)

3. [Kim SW, Jeong EJ, Kang HS, Tak JI, Bang WY, Heo JB, Jeong JY, Yoon GM, Kang HY, Bahk JD. 2006. Role of RepB in the replication of plasmid pJB01 isolated from Enterococcus faecium JC1. Plasmid 55:99–113.](http://paperpile.com/b/BW0bbP/LeyPh)

4. [Francia MV, Clewell DB. 2002. Amplification of the Tetracycline Resistance Determinant of pAMα1 in Enterococcus faecalis Requires a Site-Specific Recombination Event Involving Relaxase. J Bacteriol 184:5187–5193.](http://paperpile.com/b/BW0bbP/sl70P)

5. [Clewell DB, Weaver KE, Dunny GM, Coque TM, Francia MV, Hayes F. 2014. Extrachromosomal and Mobile Elements in Enterococci: Transmission, Maintenance, and Epidemiology. Massachusetts Eye and Ear Infirmary.](http://paperpile.com/b/BW0bbP/o19PW)

6. [Huo W, Adams HM, Trejo C, Badia R, Palmer KL. 2019. A Type I Restriction-Modification System Associated with Enterococcus faecium Subspecies Separation. Appl Environ Microbiol 85.](http://paperpile.com/b/BW0bbP/71OAF)

7. [Fernández-García L, Blasco L, Lopez M, Bou G, García-Contreras R, Wood T, Tomas M. 2016. Toxin-Antitoxin Systems in Clinical Pathogens. Toxins 8.](http://paperpile.com/b/BW0bbP/CthLD)

8. [Soheili S, Ghafourian S, Sekawi Z, Neela VK, Sadeghifard N, Taherikalani M, Khosravi A, Ramli R, Hamat RA. 2015. The mazEF toxin-antitoxin system as an attractive target in clinical isolates of Enterococcus faecium and Enterococcus faecalis. Drug Des Devel Ther 9:2553–2561.](http://paperpile.com/b/BW0bbP/Al9K2)

9. [Dy RL, Przybilski R, Semeijn K. 2014. A widespread bacteriophage abortive infection system functions through a Type IV toxin–antitoxin mechanism. Nucleic acids.](http://paperpile.com/b/BW0bbP/61xqz)

10. [O’Connor L, Tangney M, Fitzgerald GF. 1999. Expression, regulation, and mode of action of the AbiG abortive infection system of lactococcus lactis subsp. cremoris UC653. Appl Environ Microbiol 65:330–335.](http://paperpile.com/b/BW0bbP/Dsvjn)

11. [Hasman H. 2005. The tcrB gene is part of the tcrYAZB operon conferring copper resistance in Enterococcus faecium and Enterococcus faecalis. Microbiology 151:3019–3025.](http://paperpile.com/b/BW0bbP/JXJ08)

12. [Lin J. 2014. Antibiotic growth promoters enhance animal production by targeting intestinal bile salt hydrolase and its producers. Front Microbiol 5:33.](http://paperpile.com/b/BW0bbP/4ar3R)

13. [Nilsson O, Myrenås M, Ågren J. 2016. Transferable genes putatively conferring elevated minimum inhibitory concentrations of narasin in Enterococcus faecium from Swedish broilers. Vet Microbiol 184:80–83.](http://paperpile.com/b/BW0bbP/AWQUH)

14. [Haas W, Sublett J, Kaushal D, Tuomanen EI. 2004. Revising the role of the pneumococcal vex-vncRS locus in vancomycin tolerance. J Bacteriol 186:8463–8471.](http://paperpile.com/b/BW0bbP/68vCp)

15. [Rusniok C, Couvé E, Da Cunha V, El Gana R, Zidane N, Bouchier C, Poyart C, Leclercq R, Trieu-Cuot P, Glaser P. 2010. Genome sequence of Streptococcus gallolyticus: insights into its adaptation to the bovine rumen and its ability to cause endocarditis. J Bacteriol 192:2266–2276.](http://paperpile.com/b/BW0bbP/Hfypr)

16. [Kurushima J, Ike Y, Tomita H. 2016. Partial Diversity Generates Effector Immunity Specificity of the Bac41-Like Bacteriocins of Enterococcus faecalis Clinical Strains. J Bacteriol 198:2379–2390.](http://paperpile.com/b/BW0bbP/fsSUF)

17. [Antipov D, Hartwick N, Shen M, Raiko M, Pevzner PA. 2016. plasmidSPAdes : Assembling Plasmids from Whole Genome Sequencing Data. Bioinformatics 32:3380–3387.](http://paperpile.com/b/BW0bbP/fNwr8)

18. [Seemann T. 2014. Prokka: Rapid prokaryotic genome annotation. Bioinformatics 30:2068–2069.](http://paperpile.com/b/BW0bbP/R5NFc)

19. [Page AJ, Cummins CA, Hunt M, Wong VK, Reuter S, Holden MTG, Fookes M, Falush D, Keane JA, Parkhill J. 2015. Roary: rapid large-scale prokaryote pan genome analysis. Bioinformatics 31:3691–3693.](http://paperpile.com/b/BW0bbP/Ahu3k)

20. [Maaten L van der, Hinton G. 2008. Visualizing Data using t-SNE. J Mach Learn Res 9:2579–2605.](http://paperpile.com/b/BW0bbP/3Gjne)

21. [Krijthe J. 2015. Rtsne: T-Distributed Stochastic Neighbor Embedding using Barnes-Hut Implementation (R package version 0.10). Computer Software.](http://paperpile.com/b/BW0bbP/ow7DZ)

22. [Clewell DB, Weaver KE, Dunny GM, Coque TM, Francia MV, Hayes F. 2014. Extrachromosomal and Mobile Elements in Enterococci: Transmission, Maintenance, and Epidemiology.](http://paperpile.com/b/BW0bbP/GiXNg)

23. [Wick RR, Judd LM, Gorrie CL, Holt KE. 2017. Unicycler: Resolving bacterial genome assemblies from short and long sequencing reads. PLoS Comput Biol 13:e1005595.](http://paperpile.com/b/BW0bbP/orEgO)

24. [Bankevich A, Nurk S, Antipov D, Gurevich A a., Dvorkin M, Kulikov AS, Lesin VM, Nikolenko SI, Pham S, Prjibelski AD, Pyshkin AV, Sirotkin AV, Vyahhi N, Tesler G, Alekseyev M a., Pevzner P a. 2012. SPAdes: A New Genome Assembly Algorithm and Its Applications to Single-Cell Sequencing. J Comput Biol 19:455–477.](http://paperpile.com/b/BW0bbP/xs5Pb)

25. [Walker BJ, Abeel T, Shea T, Priest M, Abouelliel A, Sakthikumar S, Cuomo CA, Zeng Q, Wortman J, Young SK, Earl AM. 2014. Pilon: an integrated tool for comprehensive microbial variant detection and genome assembly improvement. PLoS One 9:e112963.](http://paperpile.com/b/BW0bbP/0gumb)

26. [Jensen LB, Garcia-Migura L, Valenzuela AJS, Løhr M, Hasman H, Aarestrup FM. 2010. A classification system for plasmids from enterococci and other Gram-positive bacteria. J Microbiol Methods 80:25–43.](http://paperpile.com/b/BW0bbP/QkWvX)

27. [Ondov BD, Treangen TJ, Melsted P, Mallonee AB, Bergman NH, Koren S, Phillippy AM. 2016. Mash: fast genome and metagenome distance estimation using MinHash. Genome Biol 17:132.](http://paperpile.com/b/BW0bbP/HtwJD)

28. [Yu G, Smith DK, Zhu H, Guan Y, Lam TT-Y. 2017. ggtree : an r package for visualization and annotation of phylogenetic trees with their covariates and other associated data. Methods Ecol Evol 8:28–36.](http://paperpile.com/b/BW0bbP/AJqjG)
